# Genome-wide association studies identify novel genetic loci for epigenetic age acceleration among survivors of childhood cancer

**DOI:** 10.1186/s13073-022-01038-6

**Published:** 2022-03-22

**Authors:** Qian Dong, Nan Song, Na Qin, Cheng Chen, Zhenghong Li, Xiaojun Sun, John Easton, Heather Mulder, Emily Plyler, Geoffrey Neale, Emily Walker, Qian Li, Xiaotu Ma, Xiang Chen, I-Chan Huang, Yutaka Yasui, Kirsten K. Ness, Jinghui Zhang, Melissa M. Hudson, Leslie L. Robison, Zhaoming Wang

**Affiliations:** 1 Department of Epidemiology and Cancer Control, St. Jude Children’s Research Hospital, 262 Danny Thomas Place, MS 735, Memphis, TN 38105 USA; 2 College of Pharmacy, Chungbuk National University, Cheongju, Korea; 3 Department of Epidemiology, Center for Global Health, School of Public Health, Nanjing Medical University, Nanjing, Jiangsu China; 4 School of Public Health, Shanghai Jiaotong University, Shanghai, China; 5 Department of Structural Biology, St. Jude Children’s Research Hospital, Memphis, TN USA; 6 Department of Computational Biology, St. Jude Children’s Research Hospital, Memphis, TN USA; 7 Hartwell Center, St. Jude Children’s Research Hospital, Memphis, TN USA; 8 Department of Biostatistics, St. Jude Children’s Research Hospital, Memphis, TN USA; 9 Department of Oncology, St. Jude Children’s Research Hospital, Memphis, TN USA

**Keywords:** Genome-wide association study, Epigenetic age acceleration, Childhood cancer, Survivorship, *SELP*, *HLA*

## Abstract

**Background:**

Increased epigenetic age acceleration (EAA) in survivors of childhood cancer is associated with specific treatment exposures, unfavorable health behaviors, and presence of certain chronic health conditions. To better understand inter-individual variability, we investigated the genetic basis underlying EAA.

**Methods:**

Genome-wide association studies of EAA based on multiple epigenetic clocks (Hannum, Horvath, PhenoAge, and GrimAge) were performed. MethylationEPIC BeadChip array and whole-genome sequencing data were generated with blood-derived DNA from participants in the St. Jude Lifetime Cohort Study (discovery: 2138 pre-existing and 502 newly generated data, all survivors; exploratory: 282 community controls). Linear regression models were fit for each epigenetic age against the allelic dose of each genetic variant, adjusting for age at sampling, sex, and cancer treatment exposures. Fixed-effects meta-analysis was used to combine summary statistics from two discovery data sets. LD (Linkage disequilibrium) score regression was used to estimate single-nucleotide polymorphism (SNP)-based heritability.

**Results:**

For EAA-Horvath, a genome-wide significant association was mapped to the *SELP* gene with the strongest SNP rs732314 (meta-GWAS: *β*=0.57, *P*=3.30×10^-11^). Moreover, the stratified analysis of the association between rs732314 and EAA-Horvath showed a substantial heterogeneity between children and adults (meta-GWAS: *β*=0.97 vs. 0.51, *I*^*2*^=73.1%) as well as between survivors with and without chest/abdominal/pelvic-RT exposure (*β*=0.64 vs. 0.31, *I*^*2*^=66.3%). For EAA-Hannum, an association was mapped to the *HLA* locus with the strongest SNP rs28366133 (meta-GWAS: *β*=0.78, *P*=3.78×10^-11^). There was no genome-wide significant hit for EAA-PhenoAge or EAA-GrimAge. Interestingly, among community controls, rs732314 was associated with EAA-Horvath (*β*=1.09, *P*=5.43×10^-5^), whereas rs28366133 was not associated with EAA-Hannum (*β*=0.21, *P*=0.49). The estimated heritability was 0.33 (SE=0.20) for EAA-Horvath and 0.17 (SE=0.23) for EAA-Hannum, but close to zero for EAA-PhenoAge and EAA-GrimAge.

**Conclusions:**

We identified novel genetic variants in the *SELP* gene and *HLA* region associated with EAA-Horvath and EAA-Hannum, respectively, among survivors of childhood cancer. The new genetic variants in combination with other replicated known variants can facilitate the identification of survivors at higher risk in developing accelerated aging and potentially inform drug targets for future intervention strategies among vulnerable survivors.

**Supplementary Information:**

The online version contains supplementary material available at 10.1186/s13073-022-01038-6.

## Background

A 5-year survival for children diagnosed with cancer has increased to ~85% due to remarkable progress in treatment [1]. Thus, the population of childhood cancer survivors has grown rapidly and is estimated to exceed 500,000 in the USA [2]. Survivors of childhood cancer experience accelerated aging [3, 4], which may not only correlate with increased risk of chronic health conditions (CHCs), but likely reflect the influences of genetics, treatment/environmental exposures, and behavioral factors that, in combination, impact physiological health [5, 6]. Understanding biological pathways and determinants underpinning the aging process among childhood cancer survivors will facilitate the identification of individuals at the greatest risk and inform molecular candidates and biological processes for those who may benefit from targeted therapies soon after curative treatment or later among long-term survivors.

Several molecular biomarkers of aging are available [5–10], including DNA methylation (DNAm)-based epigenetic age. Epigenetic age acceleration (EAA) (i.e., the difference between epigenetic and chronological ages) demonstrates excellent predictive accuracy for physiological aging and age-related disease risks in the general population [11]. We recently reported that EAA based on PhenoAge (i.e., Levine’s clock) [12] is statistically significantly higher in survivors of childhood cancer than community controls and is associated with specific treatment exposures, unfavorable health behaviors, and presence of different CHCs such as hypertension, myocardial infarction, obesity, obstructive pulmonary deficit, and peripheral sensory neuropathy [6]. However, the genetic basis underpinning EAA has not yet been investigated among the childhood cancer survivors.

A meta-analysis of genome-wide association studies (GWASs) on 9907 individuals demonstrated that the EAA derived from various epigenetic clocks is a trait with a moderate heritability (*h*^*2*^=0.19) in the general population [13] and identified five loci associated with IEAA-Horvath (Intrinsic Epigenetic Age Acceleration) and three associated with EEAA-Hannum (Extrinsic Epigenetic Age Acceleration) [13]. Gilson et al. performed single-nucleotide polymorphism (SNP)-based and gene-based GWAS of both IEAA-Horvath and EEAA-Hannum on 13,493 individuals of European ancestry and identified 10 independent SNPs and 21 genes associated with IEAA-Horvath including the notable *PIK3CB* related to human longevity, and one SNP and 12 genes associated with EEAA-Hannum including *CISD2* related to lifespan control [14]. Another large-scale GWAS study comprised of more than 40,000 individuals identified a bulk of 137 loci associated with DNAm biomarkers of aging [15], which enhanced knowledge about the genetic architecture underlying EAA. In addition to germline variants, clonal hematopoiesis of indeterminant potentials (CHIP), which were somatically acquired genetic factors and predictive of the development of leukemia (particularly, acute myeloid leukemia in the elderly) [16], were associated with EAA derived from multiple epigenetic clocks [17].

Thus, to advance the understanding about the genetic factors underlying EAA among childhood cancer survivors, we performed GWAS with Infinium MethylationEPIC BeadChip array and whole-genome sequencing (WGS) data generated with blood-derived DNA from participants of the St. Jude Lifetime Cohort Study (SJLIFE) [18, 19]. Specifically, we aimed to search for novel genetic loci, evaluate previous findings reported in the general population, and characterize genetic contributions to EAA in childhood cancer survivors.

## Methods

### Study population

SJLIFE participants (*n*=2922) with pre-exiting whole-genome sequencing data [20, 21] were included in the current study. The first discovery data set, denoted as SJLIFE1 Survivors, included 2138 survivors previously scanned with EPIC array [6]. The second discovery data set, denoted as SJLIFE2 survivors, included 502 survivors newly scanned with EPIC array in an expansion study focused primarily on childhood and adolescent survivors. The third exploratory data set, denoted Community Controls, included 282 controls with no history of childhood cancer, who were enrolled in SJLIFE study and frequency matched to the survivors by age, sex, and race. This set of controls was also previously scanned with an EPIC array [6].

### Demographic, diagnostic, and treatment data

Demographic characteristics (sex and race/ethnicity) and clinical information (primary diagnosis, age at diagnosis, and treatment exposures) were abstracted using a structured protocol [19]. Region-specific radiotherapy (RT) dosimetry, including brain-RT, chest-RT, and abdomen/pelvic-RT, were estimated using radiation oncology treatment records [19]. Cumulative doses of individual chemotherapeutic agents, including alkylating agents, anthracyclines, epipodophyllotoxins, glucocorticoids, platinum, and vincristine, were abstracted from medical records.

### DNA methylation measurement

Genome-wide methylation data were generated using Infinium MethylationEPIC BeadChip array on whole blood-derived DNA. Laboratory work followed the standard procedure as described previously [6, 21], including DNA extraction, bisulfite treatment, array hybridization, and scanning. The raw intensity was exported from Illumina Genome Studio and analyzed in R (version 3.6.3) using the minfi package [22]. Detailed quality controls (QCs) and data normalization were described previously [23]. After QCs, the data set comprised beta-values for 689,419 CpGs.

### Epigenetic age and epigenetic age acceleration

Epigenetic age estimates based on different clocks, including those of Horvath [24], Hannum [25], PhenoAge [12], and GrimAge [26], were obtained from the online New Methylation Age Calculator (https://dnamage.genetics.ucla.edu/new) [24]. Normalized DNA methylation beta-values and the sample annotation files were submitted to the calculator, using the “Advanced Analysis” option. Blood cell abundance measures were also estimated by the calculator, based on DNA methylation levels, as described previously [27, 28]. EAAs were estimated as residuals from a linear regression model of the estimated epigenetic age against the chronological age (i.e., age at DNA sampling). Additionally, another variation of EAA was estimated based on the Horvath clock with adjustments for leukocyte subtype proportions, commonly referred to as IEAA, which captures cellular intrinsic DNAm changes [29, 30]. In contrast, EEAA, which tracks age-related changes in blood cell composition and cellular intrinsic DNAm level [29, 30], was calculated as residuals from regressing BioAge4HAStatic (Hannum clock with up-weighting of blood cell counts) against the chronological age.

### Genotyping based on whole-genome sequencing (WGS)

WGS data (*n*=2922) for this study were obtained from a previous large effort to sequence blood-derived DNA from 4402 SJLIFE survivors as previously described [20, 21], including the first set of 3006 survivors sequenced by using HiSeq X Ten System with 36.8-fold average genome-wide coverage per sample, and the second set of 1396 survivors sequenced on Illumina NovaSeq with similar (38.7-fold) average genome-wide coverage. Sequencing reads were aligned to the GRCh38 human reference genome assembly with BWA (v0.7.12-r1039) [31] using default settings, and variant calls were processed with the GATK v3.4 pipeline by following its recommended best practices including VQSR (variant quality score recalibration) filtering [32]. The entire collection of WGS data for 4402 survivors, including raw sequence reads, aligned BAM files, and joint genotype calls (gVCFs), is accessible through St. Jude Cloud (https://stjude.cloud). Additional quality control was performed when genotypes for the subset of survivors (*n*=2640 also with DNA EPIC array data available) were extracted with VCFtools v0.1.15 [33], including the following criteria for keeping the variants or genotypes: (1) minimum genotype quality score of 20, (2) minimum depth of 5, (3) minimum mean depth of 10, (4) Hardy Weinberg Equilibrium (HWE) *P* > 1×10^-6^, (5) maximum of missing rate is 10% across all samples, and (6) minor allele frequency (MAF) > 0.01. Genotypes for a set of community controls (*n*=282) was extracted from the existing WGS data and processed in the same manner. A total of 8.3 million autosomal single-nucleotide variants (SNVs) and small insertions and deletions (indels) were advanced for further association analysis.

### Statistical and bioinformatic analyses

Population characteristics were compared between survivors in the SJLIFE1 and SJLIFE2 data sets, and between survivors and controls by chi-square test for categorical variables and *t* test for continuous variables. Pearson’s correlation coefficient (*r*) was used to measure the linear correlation between the estimated epigenetic age and the chronological age, and between a pair of the estimated epigenetic ages or EAAs based on different epigenetic clocks among survivors. For EAA GWAS, linear regression models were fit for each estimated epigenetic age against the additive dose of each genetic variant, adjusting for age at DNA sampling, sex, and cancer treatment exposures. EAA GWAS analysis was carried out for each of three data sets with both WGS and EPIC array data, respectively. Fixed-effects meta-analysis was used to combine the summary statistics of two discovery data sets. A fixed meta-GWAS *P* value threshold of 5×10^-8^ was set as the level of genome-wide significance. PLINK (1.90b) was used for the genetic association analysis [34]. Genetic heterogeneity between data sets was assessed by using *I*^*2*^ and *P* value (*P*_het_) calculated from the Cochran’s *Q* statistic. Differentially methylated regions (DMR) between survivors and community controls were analyzed with DMRcate R package [35] using R 4.0.2 [30]. Other statistical analyses were performed with R 3.6.1 [36], and a two-sided *P* value <0.05 was considered as statistically significant.

Manhattan plots were generated using CMplot [37]. Regional SNP association results were visualized with the LocusZoom [38]. All linkage disequilibrium (LD) estimates were calculated using individuals of European ancestry from the 1000 genomes reference panel using LDlink [39]. LD score regression [40] was used to estimate SNP-based heritability (*h*^*2*^ and its standard error [SE]) and to calculate genetic correlations between EAA and other traits (*n*=855) through LD Hub [41].

## Results

### Characteristics of the study population

Demographics, primary diagnosis, and treatment information for the SJLIFE1 data set were described previously [6] and were included in Table 1, along with information for the SJLIFE2 data set with newly generated DNAm data. Among those in the SJLIFE2 data set, 53.2% were male and 97.8% were non-Hispanic. Primary diagnoses were leukemia (35.1%), lymphoma (7.2%), sarcoma (7.8%), central nervous system (CNS) tumors (16.5%), and other solid tumors (33.4%). Regarding treatments, 29.3% were exposed to brain-RT, 16.3% chest-RT, 16.1% abdominal/pelvic-RT, 51.2% alkylating agents, 51.2% anthracyclines, 33.1% epipodophyllotoxins, 39.6% glucocorticoids, 25.1% platinum, and 68.3% vincristine. The median ages at diagnosis and at DNA sampling were 3.1 (range= 0.0–19.9) and 16.5 (range= 7.3–66.6) years, respectively. Among the 282 noncancer controls, 48.6% were male and 2.1% Hispanic. The median age at DNA sampling was 35.0 (range = 18.7 to 70.2) years [6].

**Table 1:** Characteristics of participants included in the study

Characteristics	SJLIFE1 survivors No. (%)	SJLIFE2 survivors No. (%)	Non-cancer controls No. (%)	SJLIFE1 vs. SJLIFE2 *P*	Survivors vs. controls *P*
Total	2138^a^	502	282		
Sex					
Male	1132 (53.0)	267 (53.2)	137 (48.6)	0.17	0.19
Female	1006 (47.0)	235 (46.8)	145 (51.4)		
Ethnicity					
Hispanic	24 (1.1)	11 (2.2)	6 (2.1)	0.18	0.15
Non-Hispanic	2114 (98.9)	491 (97.8)	276 (97.9)		
Diagnosis					
Leukemia	731 (34.2)	176 (35.1)			
Acute lymphoblastic leukemia	671 (31.4)	159 (31.7)	–		
Acute myeloid leukemia	58 (2.7)	15 (3.0)	–		
Other leukemia	2 (0.1)	2 (0.4)	–		
Lymphoma	460 (21.5)	36 (7.2)			
Hodgkin lymphoma	296 (13.8)	11 (2.2)	–		
Non-Hodgkin lymphoma	164 (7.7)	25 (5.0)	–		
Sarcoma	283 (13.2)	39 (7.8)	–		
Ewing sarcoma	76 (3.6)	7 (1.4)	–		
Osteosarcoma	76 (3.6)	5 (1.0)			
Rhabdomyosarcoma	73 (3.4)	15 (3.0)	–		
Nonrhabdomyosarcoma	58 (2.7)	12 (2.4)	–		
CNS tumors	245 (11.5)	83 (16.5)			
Astrocytoma or glioma	113 (5.3)	41 (8.2)	–		
Medulloblastoma or PNET	59 (2.8)	15 (3.0)	–		
Ependymoma	27 (1.3)	8 (1.6)	–		
Other CNS tumors	46 (2.2)	19 (3.8)	–		
Embryonal	285 (13.3)	82 (16.3)			
Wilms tumor	140 (6.5)	32 (6.4)	–		
Neuroblastoma	109 (5.1)	39 (7.8)	–		
Germ cell tumor	36 (1.9)	11 (2.2)	–		
Other	134 (6.3)	86 (17.1)			
Retinoblastoma	50 (2.3)	67 (13.3)	–		
Hepatoblastoma	15 (0.7)	2 (0.4)	–		
Melanoma	13 (0.6)	4 (0.8)	–		
Carcinomas	24 (1.1)	3 (0.6)	–		
Others	32 (1.5)	10 (2.0)	–		
Radiation					
Brain RT	666 (31.2)	147 (29.3)	–		
Chest RT	608 (28.4)	82 (16.3)	–		
Abdomen/pelvis RT	479 (22.4)	81 (16.1)	–		
Chemotherapy					
Alkylating agent	1269 (59.4)	257 (51.2)	–		
Anthracyclines	1265 (59.2)	257 (51.2)	–		
Epipodophyllotoxins	762 (35.6)	166 (33.1)	–		
Glucocorticoids	1010 (47.2)	199 (39.6)	–		
Platinum	271 (12.7)	126 (25.1)	–		
Vincristine	1489 (69.6)	343 (68.3)	–		
Median age at diagnosis, years (range)	7.3 (0.0, 23.6)	3.1 (0.0, 19.9)	NA		
Median age at DNA sampling, years (range)	31.8 (6.0, 66.4)	16.5 (7.3, 66.6)	35.0 (18.7, 70.2)	<0.001	<0.001

*Abbreviations*: *SJLIFE1 survivors* The first discovery data set of 2138 survivors included in our previously published study (ref. [6]), *SJLIFE2 survivors* The second discovery data set of 502 children and adolescent survivors, *CNS* Central nervous system, *PNET* Primitive neuroectodermal tumor, *RT* Radiotherapy

^a^Starting from 2139 survivors included in our previous publication (ref. [6]), a retinoblastoma survivor failed the minfi QC with low total intensity and hence was excluded from this current analysis

### Epigenetic age and epigenetic age acceleration

**Fig. 1:**
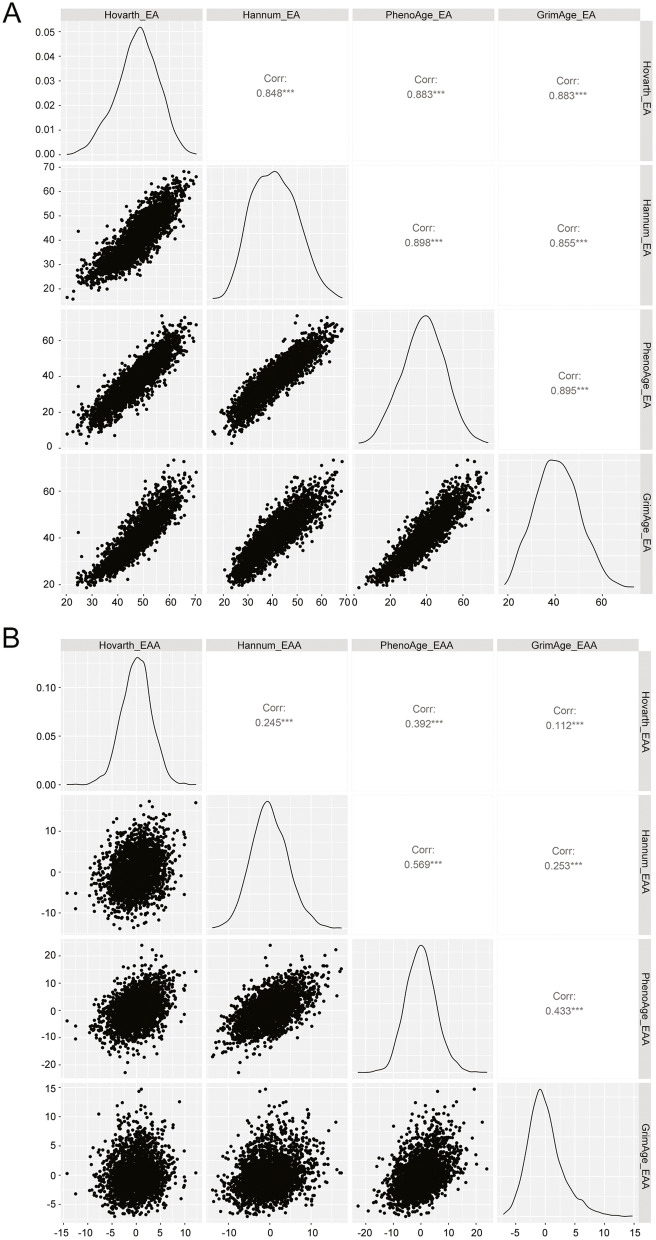
Pairwise correlations of four epigenetic age (**A**) and epigenetic age acceleration (**B**). Abbreviations: epigenetic age (EA) and epigenetic age acceleration (EAA). Pair-wise Pearson correlation coefficients were shown with *P*< 0.001 denoted as ***

All four epigenetic age estimates had high Pearson’s correlation with chronological age (*r*=0.87–0.95) (Additional file 1: Fig. S1). Pair-wise comparisons among the four epigenetic age estimates also showed a high correlation (*r*=0.85–0.90) (Fig. 1A). However, the pair-wise comparison among the four EAA showed a range of correlations (*r*=0.11–0.57); the pair of EAA-Hannum and EAA-PhenoAge had a moderately strong correlation (*r*=0.57), whereas EAA-Horvath and EAA-GrimAge had weak correlation (*r*=0.11) (Fig. 1B).

### Genome-wide association analysis of EAA-Horvath

**Fig. 2:**
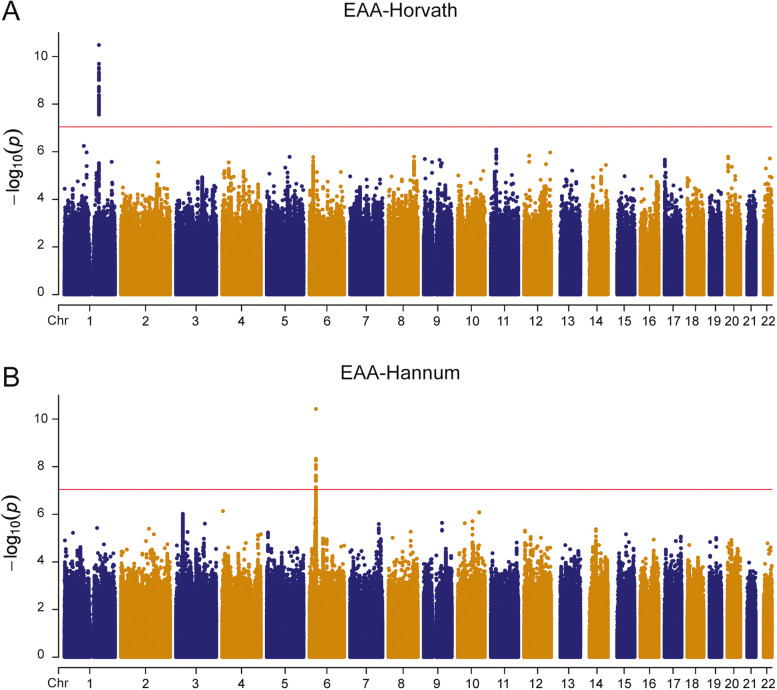
Manhattan plots of genome-wide association study (GWAS) for epigenetic age acceleration (EAA)-Horvath (**A**) and EAA-Hannum (**B**). ^a^Each dot represents the test result for one SNP. ^b^*X*-axis is the genomic location along each chromosome, and *Y*-axis is -log10 of *P* value. ^c^The red horizontal line corresponds to the *P* value of 5×10^-8^

**Fig. 3:**
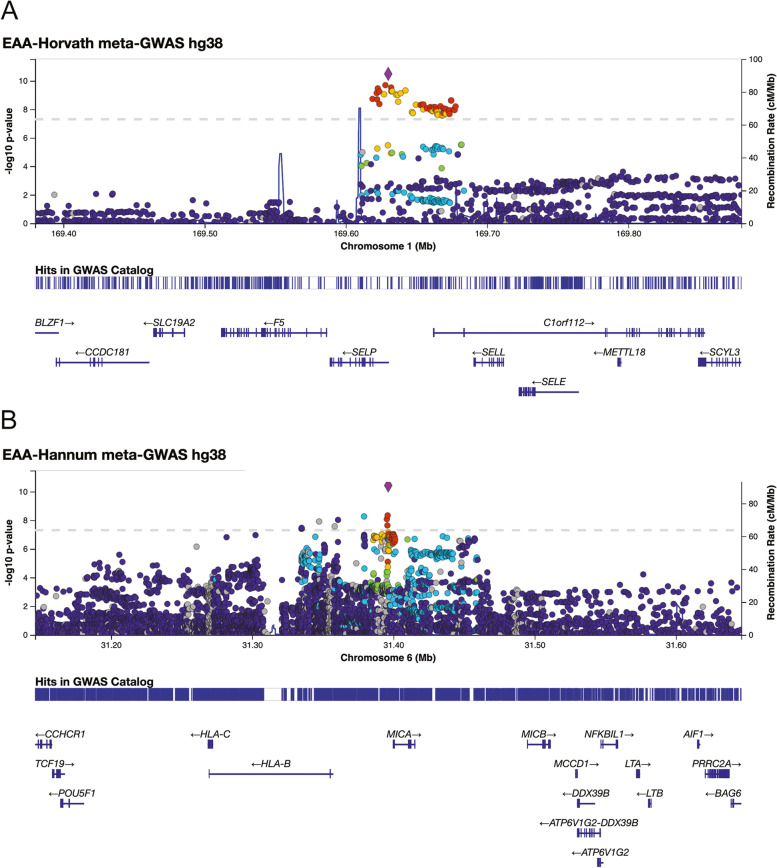
Regional plots for genetic associations between *SELP* locus and epigenetic age acceleration (EAA)-Horvath (**A**), *HLA* locus, and EAA-Hannum (**B**). ^a^Each dot represents the test result for one SNP. ^b^*X*-axis is the genomic location along each chromosome, and *Y*-axis is -log10 of *P* value. ^c^The red horizontal line corresponds to the *P* value of 5×10^-8^. ^d^SNP depicted in diamond is the index SNP for the region, and all other SNPs were depicted as circles and their correlations (i.e., pair-wise linkage disequilibrium) with the index SNP was warm/cool color-coded

The overall association results (-log_10_ of *P* values) for the EAA based on the Horvath clock [24] in the meta-GWAS of two discovery data sets (SJLIFE1 and SJLIFE2) with a total of 2640 survivors were illustrated in Fig. 2A, with a genomic inflation factor of 1.01, suggesting little systematic inflation (Additional file 1: Fig. S2A). The strongest association was observed for rs732314 (combined: *β*=0.57, *P*=3.30×10^-11^, *I*^2^=54.36%; SJLIFE1: *β*=0.50, *P*=1.55×10^-7^; SJLIFE2: *β*=0.82, *P*=2.09×10^-5^), which is mapped to the first intron of *SELP* gene on chromosome 1, and this SNP also showed statistically significant association among the community controls (Table 2). There were 53 other variants reaching genome-wide significance (*P*<5×10^-8^) (Additional file 1: Table S1), and they are all mapped to the same genomic region with high LD with the index variant rs732314 (*R*^2^ > 0.62) based on CEU (Utah residence from North and West Europe) population in 1000 Genomes project (Fig. 3A).

**Table 2:** Top SNP significantly associated with EAA-Horvath and EAA-Hannum among survivors (SJLIFE1, SJLIFE2) and controls

GWAS	SNP	Chr	Pos_hg38	Effect allele	Other allele	Population	Effect size	(SE)	*P*	*P*_het_	*I*^2^ (%)
EAA-Horvath	rs732314	1	169630016	C	T	SJLIFE1 survivors	0.50	0.10	1.55E−07		
						SJLIFE2 survivors	0.82	0.19	2.09E−05		
						Combined survivors	0.57	0.09	3.30E−11	0.14	54.36
						Community controls	1.09	0.27	5.43E−05		
EAA-Hannum	rs28366133	6	31396299	C	T	SJLIFE1 survivors	0.76	0.14	5.30E−08		
						SJLIFE2 survivors	0.84	0.23	2.05E−04		
						Combined survivors	0.78	0.12	3.78E−11	0.75	0.00
						Community controls	0.21	0.30	4.88E−01		

*Abbreviations*: *GWAS* Genome-wide association study, *EAA* Epigenetic age acceleration, *Chr* Chromosome, *SE* Standard error, *SJLIFE1 survivors* The first discovery data set of 2138 survivors included in our previously published study (ref. [6]), *SJLIFE2 survivors* The second discovery data set of 502 children and adolescent survivors, *Combined survivors* A combined set (meta-GWAS analysis) of the two discovery data sets (SJLIFE1 and SJLIFE2), *Community controls* A set of 282 community controls

In addition, we carried out an analysis with another variation of EAA based on the Horvath clock, i.e., IEAA [29]. The top findings were similar, but with one additional SNP rs3917679 reaching the genome-wide significance level (Additional file 1: Table S2). This SNP, which also mapped to the first intron of *SELP*, had high LD with rs732314 (*R*^2^=0.98) and perfect LD (*R*^2^=1) with rs3917647, suggesting that they share the same haplotype and presumably tag the same causative variant.

### Genome-wide association analysis of EAA-Hannum

A similar analysis was performed for the EAA based on the Hannum clock [25]. The overall association results (-log_10_ of *P* values) across the genome in the meta-GWAS were provided in Fig. 2B. The genomic inflation factor was 1.02, suggesting little systematic inflation (Additional file 1: Fig. S2B). The most strongly associated SNP was rs28366133 (combined: *β*=0.78, *P*=3.78×10^-11^, *I*^*2*^=0; SJLIFE1: *β*=0.76, *P*=5.30×10^-8^; SJLIFE2: *β*=0.84, *P*=2.05×10^-4^), but this SNP did not show a statistically significant association among the community controls (Table 2). There were 10 other variants reaching genome-wide significance (Additional file 1: Table S1), and they were all mapped to the complex *HLA* region with a number of genes nearby and moderate to high LD with the index variant rs28366133 (*R*^2^ > 0.29) (Fig. 3B). To explore reasons for the heterogeneity, we analyzed and observed significant DMRs in the *HLA* locus between survivors and community controls (Additional file 1: Table S3). In further analysis conditioning on rs28366133, the strength of association with these ten variants was attenuated with the lowest *P* value >1.0×10^-3^, suggesting that a secondary signal in this locus is less likely.

In addition, we analyzed another variation of EAA based on the Hannum clock, i.e., EEAA. Only rs28366133 was associated with EEAA-Hannum at the genome-wide significant level (Additional file 1: Table S2).

There was no genome-wide significant association in our analysis for EAA based on PhenoAge and GrimAge.

### Stratified analysis by chronologically defined age and sex

The fixed effects meta-analysis that showed substantial heterogeneity for the top EAA-Horvath associated SNP (*I*^*2*^: 54.36% for rs732314, Table 2) prompted us to conduct stratified analysis, given the difference in distributions of chronological age between the SJLIFE1 and SJLIFE2 data sets. Among children (<18 years old), the association between rs732314 and EAA-Horvath was stronger with a much larger effect size (combined: *β*=0.97, *P*=1.52×10^-5^, *I*^*2*^=0.0%; SJLIFE1: *β*=0.96, *P*=0.062; SJLIFE2: *β*=0.97, *P*=1.27×10^-4^) compared to adults (combined: *β*=0.51, *P*=1.39×10^-8^, *I*^*2*^=0.0%; SJLIFE1: *β*=0.51, *P*=1.14×10^-7^; SJLIFE2: *β*=0.51, *P*=0.05) (Table 3). It is notable that the effect size is highly consistent between the SJLIFE1 and SJLIFE2 data sets among children (0.96 vs. 0.97) or adults (0.51 vs. 0.51). Overall, a comparison between the children and adults showed substantial heterogeneity (combined: *β*=0.97 vs. 0.51, *I*^*2*^=73.1%, *P*_het_=0.05).

**Table 3:** Age-related, gender-, and treatment-stratified effect sizes of significant SNPs associated with EAA among different data sets

GWAS	SNP	Population	−	Effect size	(SE)	*P*	*P*_het_	*I*^2^ (%)
EAA-Horvath	rs732314	survivors (SJLIFE1) <18 Y	70	0.96	0.50	6.16E−02		
		survivors (SJLIFE2) <18 Y	314	0.97	0.25	1.27E−04		
		survivors (combined) <18 Y	384	0.97	0.22	1.52E−05	0.98	0.00
		survivors (SJLIFE1) ≥18 Y	2066	0.51	0.10	1.14E−07		
		survivors (SJLIFE2) ≥18 Y	188	0.51	0.26	5.04E−02		
		survivors (combined) ≥18 Y	2254	0.51	0.09	1.39E−08	1.00	0.00
		survivors (SJLIFE1) male	1132	0.46	0.13	3.92E−04		
		survivors (SJLIFE2) male	267	0.79	0.25	1.71E−03		
		survivors (combined) male	1399	0.53	0.11	3.94E−06	0.24	28.57
		survivors (SJLIFE1) female	1004	0.54	0.14	2.10E−04		
		survivors (SJLIFE2) female	235	0.84	0.30	5.04E−03		
		survivors (combined) female	1239	0.60	0.13	4.58E−06	0.35	0.00
		survivors (SJLIFE1) trunk-RT_Yes	649	0.37	0.17	3.47E−02		
		survivors (SJLIFE2) trunk-RT_Yes	91	0.03	0.39	9.39E−01		
		survivors (combined) trunk-RT_Yes	740	0.31	0.16	4.99E−02	0.42	0.00
		survivors (SJLIFE1) trunk-RT_No	1487	0.56	0.12	1.30E−06		
		survivors (SJLIFE2) trunk-RT_No	411	0.91	0.22	3.46E−05		
		survivors (combined) trunk-RT_No	1898	0.64	0.10	3.88E−10	0.16	49.83
EAA-Hannum	rs28366133	survivors (SJLIFE1) <18 Y	70	0.17	0.73	8.20E−01		
		survivors (SJLIFE2) <18 Y	314	1.13	0.27	5.17E−05		
		survivors (combined) <18 Y	384	1.01	0.26	8.80E−05	0.21	35.12
		survivors (SJLIFE1) ≥18 Y	2066	0.80	0.14	2.54E−08		
		survivors (SJLIFE2) ≥18 Y	188	0.28	0.40	4.78E−01		
		survivors (combined) ≥18 Y	2254	0.74	0.13	3.69E−08	0.23	32.01
		survivors (SJLIFE1) male	1131	0.74	0.19	1.40E−04		
		survivors (SJLIFE2) male	267	0.91	0.32	4.43E−03		
		survivors (combined) male	1398	0.79	0.17	1.97E−06	0.64	0.00
		survivors (SJLIFE1) female	1005	0.77	0.20	1.39E−04		
		survivors (SJLIFE2) female	235	0.71	0.33	3.47E−02		
		survivors (combined) female	1240	0.76	0.17	1.23E−05	0.87	0.00
		survivors (SJLIFE1) chest-RT_Yes	608	0.72	0.27	6.89E−03		
		survivors (SJLIFE2) chest-RT_Yes	83	0.84	0.71	2.41E−01		
		survivors (combined) chest-RT_Yes	691	0.74	0.25	3.14E−03	0.88	0.00
		survivors (SJLIFE1) chest-RT_No	1528	0.77	0.16	2.48E−06		
		survivors (SJLIFE2) chest-RT_No	419	0.88	0.24	3.02E−04		
		survivors (combined) chest-RT_No	1947	0.80	0.14	2.56E−09	0.70	0.00

*Abbreviations*: *GWAS* Genome-wide association study, *EAA* Epigenetic age acceleration, *Chr* Chromosome, *SE* Standard error, *N* Number of samples, *survivor (SJLIFE1)* The first discovery data set of 2138 survivors included in our previously published study (ref. [6]), *survivor (SJLIFE2)* The second discovery data set of 502 children and adolescent survivors, *survivors (combined)* A combined set (meta-GWAS analysis) of the two discovery data sets (SJLIFE1 and SJLIFE2), *trunk-RT* Chest/abdominal/pelvic radiation therapy, *chest-RT* Chest radiation therapy

The association between rs28366133 and EAA-Hannum in children (combined: *β*=1.01, *P*=8.80×10^-5^, *I*^*2*^=35.12%; SJLIFE1: *β*=0.17, *P*=0.82; SJLIFE2: *β*=1.13, *P*=5.17×10^-5^) did not vary much from that in adults (combined: *β*=0.74, *P*=3.69×10^-8^, *I*^*2*^=32.01%; SJLIFE1: *β*=0.80, *P*=2.54×10^-8^; SJLIFE2: *β*=0.28, *P*=0.48) (Table 3). There was only moderate heterogeneity between SJLIFE1 and SJLIFE2 data sets among children (*I*^*2*^=35.12) or adults (*I*^*2*^=32.01).

Sex was significantly associated with EAA-Horvath or EAA-Hannum in the multivariable regression models (Additional file 1: Table S4). The association between rs732314 and EAA-Horvath was slightly stronger in females (*β*=0.60 vs. 0.53) whereas the association between rs28366133 and EAA-Hannum was slightly stronger in males (*β*=0.79 vs. 0.76) (Table 3).

### Stratified analysis by treatment exposures

In the multivariable regression models, chest-RT, abdominal/pelvic-RT, and alkylators were significantly associated with EAA-Horvath, and chest-RT was significantly associated with EAA-Hannum (Additional file 1: Table S4). The association between rs732314 and EAA-Horvath was much stronger in survivors without exposure to chest/abdominal/pelvic-RT (combined: *β*=0.64 vs 0.31, *I*^*2*^=66.3%, *P*_het_=0.08) whereas the association between rs28366133 and EAA-Hannum was slightly stronger in those without exposure to chest-RT (combined: *β*=0.80 vs. 0.74) (Table 3).

### SNP-based heritability and genetic correlation with other traits

The estimated *h*^*2*^ using LD score regression was 0.33 (SE=0.20) and 0.17 (SE=0.23) for EAA-Horvath and EAA-Hannum, respectively. The point estimates were negative numbers and nearly zero for EAA-PhenoAge and EAA-GrimAge (Additional file 1: Table S5). Notably, there were significant positive genetic correlations between EAA-Horvath and heart rate, pulse rate, glycoprotein, falls in the last year, and a negative genetic correlation with creatinine (Additional file 1: Table S6).

### Previously established loci in survivors of childhood cancer

Among the 39 SNPs associated with IEAA in the general population, 20 were replicated (*P*<0.05) based on our meta-analysis of the SJLIFE1 and SJLIFE2 data sets (Additional file 1: Table S7). Similarly, only a limited number of the SNPs associated in the general population with each EAA were replicated: none of the three SNPs for EEAA-Hannum, two of the nine SNPs for EAA-Hannum, one of the four SNPs for EAA-GrimAge, and two of the 12 SNPs for EAA-PhenoAge (Additional file 1: Table S7). Notably, the previous most intriguing finding of rs2736099 (*TERT*) had a *P* value of 0.55 in our data, suggesting substantial genetic heterogeneity. We observed multiple significant DMRs between survivors and controls in the *TERT* gene including its promoter region (Additional file 1: Table S8). A polygenic risk score (PRS) for IEAA was derived from the weighted sum of the number of risk alleles (13 independent SNPs including our novel SNP rs732314) carried by each survivor. IEAA increased across the PRS quintiles with positive correlation (*r*=0.23, *P*< 2.2×10^-16^) (Additional file 1: Fig. S3), suggesting that 5.3% of the variance of IEAA can be explained by PRS.

## Discussion

It is well-established that adult survivors of childhood cancer are at risk for developing a high cumulative burden of age- and therapy-related CHCs and premature mortality, a phenomenon that might be indicative of accelerated aging [3, 4, 6, 42–48]. By leveraging the existing genetic (WGS) and epigenetic (DNAm) data in the SJLIFE cohort, an informative population of childhood cancer survivors, we performed GWAS analyses on EAA and identified two novel genome-wide significant associations. The findings identify genetic variations contributing to EAA, which may explain inter-individual variability beyond exposures of cancer treatment toxicity [6]. These novel genetic variations in combination with other replicated known hits can facilitate the identification of survivors at higher risk in accelerated aging and potentially inform drug targets for future intervention strategies in vulnerable survivors.

rs732314 was previously reported as a susceptibility locus for low high-density lipoprotein cholesterol and coronary heart disease [49]. rs732314 was also identified as a strong mQTL (methylation quantitative trait loci) with its C allele associated with a lower methylation level of cg01459453 across the human life course [50], potentially upregulating expression of the *SELP* gene (encoding P-selectin). Notably, cg01459453 was inversely correlated with chronological age and included as an aging predictive CpG in both Levine’s and Horvath’s epigenetic clocks [12, 24]. Furthermore, *SELP*, associated with IEAA-Horvath using gene-based association analysis [14], is the top-ranking aging-related gene consistently showing upregulated expression in hematopoietic stem cells across multiple studies [51]. *SELPLG* gene that encodes P-selectin glycoprotein ligand 1 had the highest level of expression in blood across all tissues analyzed in GTEx [52] and is upregulated in Alzheimer’s disease [53, 54]. We previously investigated persistent variations of DNAm-associated specific treatment exposures where cg00159243 was one of the borderline epigenome-wide significant CpGs and was inversely associated with RT exposure (raw *P* value: 8.50×10^-11^, genomic control adjusted *P* value: 2.28×10^-7^ for chest-RT, and similarly for abdominal/pelvic-RT). Specifically, survivors previously treated with chest-RT or abdominal/pelvic-RT had a low methylation level of cg00159243 and presumably had a higher expression level of the *SELPLG* gene. Moreover, cg00159243 was associated with low-grade chronic inflammation [55] and was an expression quantitative trait methylation ([eQTM], negative correlation) for *SELPLG* [56, 57]. Based on our new data and existing evidence from literature, we propose a potential molecular mechanism underlying the association between rs732314, chest/abdominal/pelvic-RT, and EAA-Horvath (Additional file 1: Fig. S4). The stratified analysis of the association between rs732314 and EAA-Horvath by age or chest/abdominal/pelvic-RT showed substantial heterogeneity between children and adults as well as between survivors with and without RT exposure. We postulate that higher expression of *SELP* (receptor) in adults or *SELPLG* (ligand) in irradiated survivors substantially weakens the association between rs732314 and EAA-Horvath.

The association between rs28366133 and EAA-Hannum association is novel and seems to be specific to the survivor population. Based on the GTEx database, rs28366133 is an expression quantitative trait locus (eQTL) for *XXbac-BPG181B23.7* (a novel transcript) and *MICA* among other genes. It is also a splicing quantitative trait locus (sQTL) for multiple genes including *MICA*, *HLA-B*, *HLA-C*, and *HLA-S* across different tissues including the blood. rs3093956 is a known hit for EAA-Hannum [15]; however, it has low LD (*r*^2^=0.075) with rs28366133. Multiple striking DMRs were observed between survivors and controls in the *HLA* region, which might be due to the fact that genotoxic cancer treatments modified the epigenome among other physiological alterations in survivors and hence altered functional genomic links (e.g., eQTL, mQTL, and eQTM) [23, 58], which may lead to either disruption or introduction of genetic associations with EAA. For the same reason, a substantial proportion (e.g., 20 out of 39 IEAA-associated SNPs) of previously reported genetic associations, including the most notable rs2736099 (*TERT*), were not replicated in our study. One caveat in interpreting DMRs in the *HLA* region is that the inferred DMRs might be partly contributed by subtle differences in genetic variations between survivors and controls in this highly polymorphic genomic region.

Although our meta-GWAS study identified genetic variants associated with EAA at a genome-wide significance level, further validation is warranted by using our expanded cohort or other survivorship cohorts in the future.

## Conclusions

In summary, we identified novel genetic variants in *SELP gene* and *HLA* region associated with EAA-Horvath and EAA-Hannum, respectively, among survivors of childhood cancer. This research represents the first EAA GWAS conducted in childhood cancer survivors, a unique clinical population who demonstrate accelerated aging. Future studies including larger and more racially and ethnically diverse populations, integrating multi-omics data such as RNA-seq and metabolomics profiling, are warranted for new discovery, association refinement, and elucidation of functional mechanisms.

## Supplementary Information

**Additional file 1: Fig. S1.** The scatter plots between chronological age and epigenetic age based on four epigenetic clocks. **Fig. S2.** QQ plots of EAA GWAS based on four epigenetic clocks. **Fig. S3.** Distribution of intrinsic epigenetic age acceleration (IEAA) across five quintiles of polygenic risk score (PRS) for IEAA. **Fig. S4.** Molecular mechanism for associations between rs732314, chest/abdominal/pelvic-RT and EAA-Horvath. **Table S1.** SNPs (except the top one) significantly associated with EAA-Horvath and EAA-Hannum among survivors (SJLIFE1, SJLIFE2) and controls. **Table S2.** Single nucleotide polymorphisms (SNPs) with significant associations with IEAA-Horvath and EEAA-Hannum among survivors (SJLIFE1, SJLIFE2) and controls. **Table S3.** Differentially methylated regions (DMR) between the SJLIFE1 data set of survivors and controls overlapping with rs28366133 (+/- 500 kb) in *HLA* region. **Table S4.** Multivariable linear regression models for the two top SNPs. **Table S5.** Estimated SNP heritability of EAA based on each of four clock methods using single-trait LD Score Regression of the GWAS of the SJLIFE1 data set. **Table S6.** Estimated genetic correlation between EAA-Horvath and other traits using LD Score Regression of the GWAS of the SJLIFE1 data set (*P*<0.05). **Table S7.** Evaluation of the known loci in the current study. **Table S8.** DMR between the survivors (SJLIFE1 data set) and controls overlapping with *TERT* gene region.

## Data Availability

The DNA methylation data, demographics, and cancer treatment information generated and analyzed in this study are accessible at the NCBI GEO website under the accession number GSE197678 (https://www.ncbi.nlm.nih.gov/geo/query/acc.cgi?acc=GSE197678) [59]. Parts of these data have been previously published in Song et al. [23] and are accessible at the NCBI GEO website under the accession number GSE169156 (https://www.ncbi.nlm.nih.gov/geo/query/acc.cgi?acc=GSE169156). Additional clinical data about the study participants in the St. Jude Lifetime Cohort can be accessed via the survivorship portal (http://survivorship.stjude.cloud/). WGS data was previously published in Qin et al. [20] and Wang et al. [21] and is accessible through the St. Jude Cloud (https://stjude.cloud) under the accession number SJC-DS-1002. To protect patients’ privacy and confidentiality (in compliance with the IRB approval and the data sharing plan), WGS data download requires an application providing a purpose and project (medical or scientific research use only), completing a data access agreement, and a subsequent approval by the data access committee. Detailed instructions can be provided by contacting support@stjude.cloud.

## Abbreviations

CHCs: Chronic health conditions
DMR: Differentially methylated regions
DNAm: DNA methylation
EAA: Epigenetic age acceleration
eQTL: Expression quantitative trait locus
eQTM: Expression quantitative trait methylation
EEAA: Extrinsic epigenetic age acceleration
GWAS: Genome-wide association study
IEAA: Intrinsic epigenetic age acceleration
LD: Linkage disequilibrium
mQTL: Methylation quantitative trait loci
QC: Quality control
RT: Radiotherapy
SNP: Single-nucleotide polymorphism
SJLIFE: St. Jude Lifetime Cohort Study
TOPMed: Trans-omics for precision medicine
WGS: Whole-genome sequencing
