# Supplementary material for: Genome-wide association studies identify novel genetic loci for epigenetic age acceleration among survivors of childhood cancer
Source: Genome Med. 2022 Mar 22;14:32. doi: 10.1186/s13073-022-01038-6 (PMC8939156; doi:10.1186/s13073-022-01038-6)
Supplement: Supplementary file 1 — Additional file 1: Fig. S1. The scatter plots between chronological age and epigenetic age based on four epigenetic clocks. Fig. S2. QQ plots of EAA GWAS based on four epigenetic clocks. Fig. S3. Distribution of intrinsic epigenetic age acceleration (IEAA) across five quintiles of polygenic risk score (PRS) for IEAA. Fig. S4. Molecular mechanism for associations between rs732314, chest/abdominal/pelvic-RT and EAA-Horvath. Table S1. SNPs (except the top one) significantly associated with EAA-Horvath and EAA-Hannum among survivors (SJLIFE1, SJLIFE2) and controls. Table S2. Single nucleotide polymorphisms (SNPs) with significant associations with IEAA-Horvath and EEAA-Hannum among survivors (SJLIFE1, SJLIFE2) and controls. Table S3. Differentially methylated regions (DMR) between the SJLIFE1 data set of survivors and controls overlapping with rs28366133 (+/- 500 kb) in HLA region. Table S4. Multivariable linear regression models for the two top SNPs. Table S5. Estimated SNP heritability of EAA based on each of four clock methods using single-trait LD Score Regression of the GWAS of the SJLIFE1 data set. Table S6. Estimated genetic correlation between EAA-Horvath and other traits using LD Score Regression of the GWAS of the SJLIFE1 data set (P<0.05). Table S7. Evaluation of the known loci in the current study. Table S8. DMR between the survivors (SJLIFE1 data set) and controls overlapping with TERT gene region. [file 13073_2022_1038_MOESM1_ESM.docx]

**Fig. S1. The scatter plots between chronological age and epigenetic age based on four epigenetic clocks.**

**Fig. S2. QQ plots of EAA GWAS based on four epigenetic clocks.** EAA, epigenetic age acceleration.

**
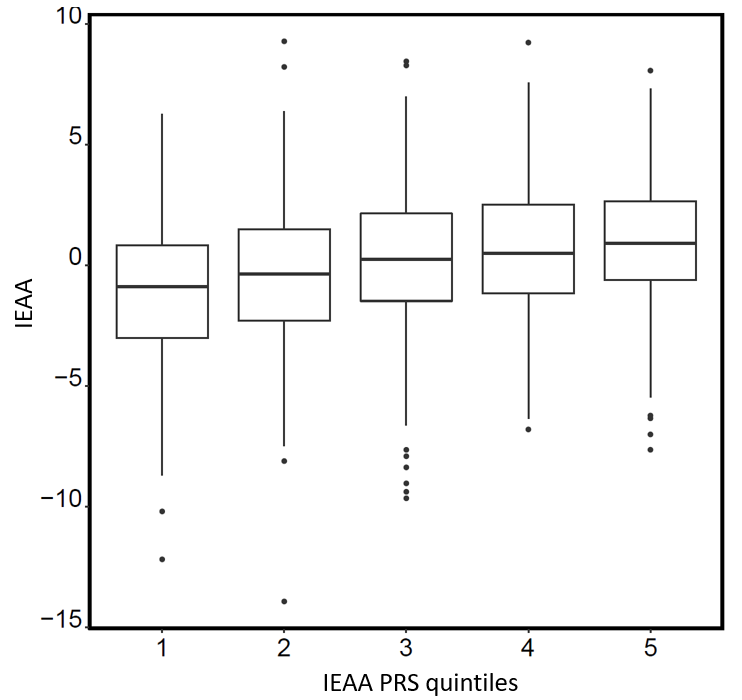
**

**Fig. S3. Distribution of intrinsic epigenetic age acceleration (IEAA) across five quintiles of polygenic risk score (PRS) for IEAA of the SJLIFE1 data set.**

**
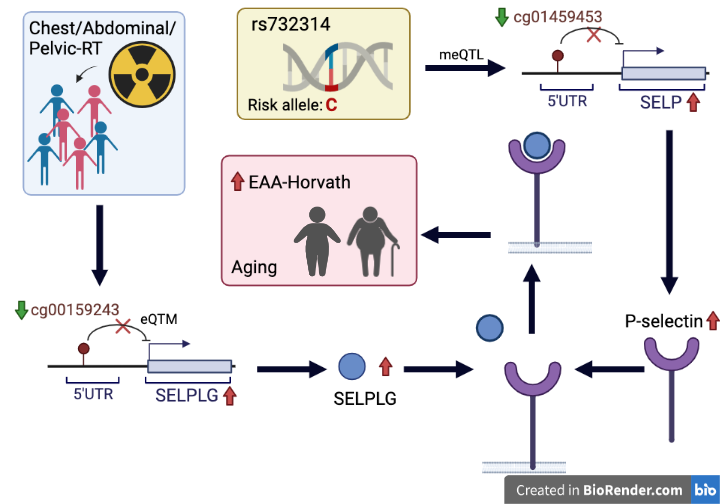
**

**Fig. S4. Molecular mechanism for associations between rs732314, chest/abdominal/pelvic-RT and EAA-Horvath.**

**Table S1. SNPs (except the top one) significantly associated with EAA-Horvath and EAA-Hannum among survivors (SJLIFE1, SJLIFE2) and controls.**

| GWAS | SNP | Chr | Pos_hg38 | Effect allele | Other allele | Population | Effect size | (SE) | *P* | *P*_het_ | I^2^ (%) |
| --- | --- | --- | --- | --- | --- | --- | --- | --- | --- | --- | --- |
| EAA-Horvath | rs3917698 | 1 | 169618879 | A | G | SJLIFE1 Survivors | 0.43 | 0.10 | 8.51E-06 |  |  |
|  |  |  |  |  |  | SJLIFE2 Survivors | 0.84 | 0.19 | 1.01E-05 |  |  |
|  |  |  |  |  |  | Combined Survivors | 0.52 | 0.09 | 1.88E-09 | 0.05 | 73.24 |
|  |  |  |  |  |  | Community Controls | 1.06 | 0.27 | 1.04E-04 |  |  |
|  | rs3917688 | 1 | 169621842 | T | C | SJLIFE1 Survivors | 0.45 | 0.10 | 2.78E-06 |  |  |
|  |  |  |  |  |  | SJLIFE2 Survivors | 0.83 | 0.19 | 1.53E-05 |  |  |
|  |  |  |  |  |  | Combined Survivors | 0.53 | 0.09 | 7.27E-10 | 0.07 | 68.87 |
|  |  |  |  |  |  | Community Controls | 1.12 | 0.27 | 3.61E-05 |  |  |
|  | rs3917683 | 1 | 169622135 | C | T | SJLIFE1 Survivors | 0.43 | 0.10 | 8.61E-06 |  |  |
|  |  |  |  |  |  | SJLIFE2 Survivors | 0.84 | 0.19 | 1.11E-05 |  |  |
|  |  |  |  |  |  | Combined Survivors | 0.51 | 0.09 | 2.03E-09 | 0.06 | 72.82 |
|  |  |  |  |  |  | Community Controls | 1.04 | 0.27 | 1.33E-04 |  |  |
|  | rs3917679 | 1 | 169622615 | T | C | SJLIFE1 Survivors | 0.47 | 0.10 | 1.16E-06 |  |  |
|  |  |  |  |  |  | SJLIFE2 Survivors | 0.82 | 0.19 | 2.24E-05 |  |  |
|  |  |  |  |  |  | Combined Survivors | 0.54 | 0.09 | 3.42E-10 | 0.10 | 62.86 |
|  |  |  |  |  |  | Community Controls | 1.08 | 0.27 | 6.03E-05 |  |  |
|  | rs3917672 | 1 | 169623743 | G | A | SJLIFE1 Survivors | 0.46 | 0.10 | 1.78E-06 |  |  |
|  |  |  |  |  |  | SJLIFE2 Survivors | 0.81 | 0.19 | 2.39E-05 |  |  |
|  |  |  |  |  |  | Combined Survivors | 0.53 | 0.09 | 5.73E-10 | 0.10 | 63.52 |
|  |  |  |  |  |  | Community Controls | 1.20 | 0.27 | 9.01E-06 |  |  |
|  | rs2236867 | 1 | 169623749 | C | A | SJLIFE1 Survivors | 0.42 | 0.10 | 1.59E-05 |  |  |
|  |  |  |  |  |  | SJLIFE2 Survivors | 0.83 | 0.19 | 1.18E-05 |  |  |
|  |  |  |  |  |  | Combined Survivors | 0.51 | 0.09 | 4.24E-09 | 0.05 | 73.75 |
|  |  |  |  |  |  | Community Controls | 1.12 | 0.27 | 4.16E-05 |  |  |
|  | rs2236866 | 1 | 169627075 | T | A | SJLIFE1 Survivors | 0.45 | 0.10 | 4.24E-06 |  |  |
|  |  |  |  |  |  | SJLIFE2 Survivors | 0.84 | 0.19 | 1.14E-05 |  |  |
|  |  |  |  |  |  | Combined Survivors | 0.53 | 0.09 | 8.91E-10 | 0.07 | 70.41 |
|  |  |  |  |  |  | Community Controls | 1.07 | 0.27 | 8.80E-05 |  |  |
|  | rs764199 | 1 | 169628000 | A | T | SJLIFE1 Survivors | 0.47 | 0.10 | 1.64E-06 |  |  |
|  |  |  |  |  |  | SJLIFE2 Survivors | 0.85 | 0.19 | 6.77E-06 |  |  |
|  |  |  |  |  |  | Combined Survivors | 0.55 | 0.09 | 2.03E-10 | 0.07 | 70.51 |
|  |  |  |  |  |  | Community Controls | 1.07 | 0.27 | 9.55E-05 |  |  |
|  | rs3917647 | 1 | 169632102 | A | G | SJLIFE1 Survivors | 0.47 | 0.10 | 8.84E-07 |  |  |
|  |  |  |  |  |  | SJLIFE2 Survivors | 0.80 | 0.19 | 2.97E-05 |  |  |
|  |  |  |  |  |  | Combined Survivors | 0.54 | 0.09 | 2.97E-10 | 0.12 | 58.03 |
|  |  |  |  |  |  | Community Controls | 1.05 | 0.27 | 1.01E-04 |  |  |
|  | rs1800807 | 1 | 169632197 | G | C | SJLIFE1 Survivors | 0.45 | 0.10 | 3.32E-06 |  |  |
|  |  |  |  |  |  | SJLIFE2 Survivors | 0.85 | 0.19 | 8.36E-06 |  |  |
|  |  |  |  |  |  | Combined Survivors | 0.53 | 0.09 | 5.42E-10 | 0.06 | 71.44 |
|  |  |  |  |  |  | Community Controls | 0.99 | 0.27 | 2.88E-04 |  |  |
|  | rs6666554 | 1 | 169632871 | G | A | SJLIFE1 Survivors | 0.46 | 0.10 | 1.68E-06 |  |  |
|  |  |  |  |  |  | SJLIFE2 Survivors | 0.80 | 0.19 | 2.69E-05 |  |  |
|  |  |  |  |  |  | Combined Survivors | 0.53 | 0.09 | 5.33E-10 | 0.12 | 59.59 |
|  |  |  |  |  |  | Community Controls | 1.00 | 0.27 | 2.55E-04 |  |  |
|  | rs2205892 | 1 | 169635516 | A | G | SJLIFE1 Survivors | 0.46 | 0.10 | 2.44E-06 |  |  |
|  |  |  |  |  |  | SJLIFE2 Survivors | 0.78 | 0.19 | 3.35E-05 |  |  |
|  |  |  |  |  |  | Combined Survivors | 0.53 | 0.09 | 9.31E-10 | 0.12 | 58.49 |
|  |  |  |  |  |  | Community Controls | 1.03 | 0.27 | 1.74E-04 |  |  |
|  | rs10800460 | 1 | 169636770 | C | T | SJLIFE1 Survivors | 0.46 | 0.10 | 2.77E-06 |  |  |
|  |  |  |  |  |  | SJLIFE2 Survivors | 0.79 | 0.19 | 2.90E-05 |  |  |
|  |  |  |  |  |  | Combined Survivors | 0.53 | 0.09 | 9.76E-10 | 0.11 | 60.67 |
|  |  |  |  |  |  | Community Controls | 1.01 | 0.27 | 2.17E-04 |  |  |
|  | rs10753788 | 1 | 169637035 | G | A | SJLIFE1 Survivors | 0.44 | 0.10 | 6.95E-06 |  |  |
|  |  |  |  |  |  | SJLIFE2 Survivors | 0.81 | 0.19 | 3.01E-05 |  |  |
|  |  |  |  |  |  | Combined Survivors | 0.52 | 0.09 | 2.94E-09 | 0.09 | 64.86 |
|  |  |  |  |  |  | Community Controls | 0.98 | 0.28 | 6.18E-04 |  |  |
|  | rs9659257 | 1 | 169638830 | G | C | SJLIFE1 Survivors | 0.46 | 0.10 | 2.15E-06 |  |  |
|  |  |  |  |  |  | SJLIFE2 Survivors | 0.79 | 0.19 | 2.95E-05 |  |  |
|  |  |  |  |  |  | Combined Survivors | 0.53 | 0.09 | 7.49E-10 | 0.12 | 59.63 |
|  |  |  |  |  |  | Community Controls | 1.03 | 0.27 | 1.56E-04 |  |  |
|  | rs6427205 | 1 | 169642234 | G | T | SJLIFE1 Survivors | 0.47 | 0.10 | 1.31E-06 |  |  |
|  |  |  |  |  |  | SJLIFE2 Survivors | 0.79 | 0.19 | 3.43E-05 |  |  |
|  |  |  |  |  |  | Combined Survivors | 0.53 | 0.09 | 4.75E-10 | 0.13 | 55.67 |
|  |  |  |  |  |  | Community Controls | 0.98 | 0.27 | 3.21E-04 |  |  |
|  | rs10800462 | 1 | 169646782 | G | A | SJLIFE1 Survivors | 0.41 | 0.10 | 2.77E-05 |  |  |
|  |  |  |  |  |  | SJLIFE2 Survivors | 0.78 | 0.19 | 3.43E-05 |  |  |
|  |  |  |  |  |  | Combined Survivors | 0.48 | 0.09 | 1.55E-08 | 0.08 | 68.17 |
|  |  |  |  |  |  | Community Controls | 1.09 | 0.27 | 6.86E-05 |  |  |
|  | rs7551819 | 1 | 169647471 | C | A | SJLIFE1 Survivors | 0.40 | 0.10 | 3.21E-05 |  |  |
|  |  |  |  |  |  | SJLIFE2 Survivors | 0.78 | 0.19 | 3.43E-05 |  |  |
|  |  |  |  |  |  | Combined Survivors | 0.48 | 0.09 | 1.86E-08 | 0.07 | 68.89 |
|  |  |  |  |  |  | Community Controls | 1.08 | 0.27 | 7.97E-05 |  |  |
|  | rs2142759 | 1 | 169649582 | G | A | SJLIFE1 Survivors | 0.43 | 0.10 | 1.01E-05 |  |  |
|  |  |  |  |  |  | SJLIFE2 Survivors | 0.78 | 0.19 | 3.43E-05 |  |  |
|  |  |  |  |  |  | Combined Survivors | 0.50 | 0.09 | 4.80E-09 | 0.09 | 64.31 |
|  |  |  |  |  |  | Community Controls | 1.08 | 0.27 | 7.97E-05 |  |  |
|  | rs6687517 | 1 | 169652823 | T | C | SJLIFE1 Survivors | -0.44 | 0.10 | 6.97E-06 |  |  |
|  |  |  |  |  |  | SJLIFE2 Survivors | -0.77 | 0.19 | 6.06E-05 |  |  |
|  |  |  |  |  |  | Combined Survivors | -0.51 | 0.09 | 5.02E-09 | 0.11 | 59.86 |
|  |  |  |  |  |  | Community Controls | -1.12 | 0.27 | 3.90E-05 |  |  |
|  | rs10800463 | 1 | 169653866 | A | G | SJLIFE1 Survivors | -0.44 | 0.10 | 6.11E-06 |  |  |
|  |  |  |  |  |  | SJLIFE2 Survivors | -0.77 | 0.19 | 6.95E-05 |  |  |
|  |  |  |  |  |  | Combined Survivors | -0.51 | 0.09 | 4.66E-09 | 0.13 | 57.21 |
|  |  |  |  |  |  | Community Controls | -1.09 | 0.27 | 6.29E-05 |  |  |
|  | rs1569473 | 1 | 169654788 | A | G | SJLIFE1 Survivors | 0.41 | 0.10 | 2.16E-05 |  |  |
|  |  |  |  |  |  | SJLIFE2 Survivors | 0.79 | 0.19 | 2.75E-05 |  |  |
|  |  |  |  |  |  | Combined Survivors | 0.49 | 0.09 | 1.01E-08 | 0.07 | 69.02 |
|  |  |  |  |  |  | Community Controls | 1.09 | 0.27 | 7.75E-05 |  |  |
|  | rs2179188 | 1 | 169654939 | A | G | SJLIFE1 Survivors | -0.44 | 0.10 | 6.26E-06 |  |  |
|  |  |  |  |  |  | SJLIFE2 Survivors | -0.77 | 0.19 | 6.57E-05 |  |  |
|  |  |  |  |  |  | Combined Survivors | -0.51 | 0.09 | 4.62E-09 | 0.12 | 58.05 |
|  |  |  |  |  |  | Community Controls | -1.08 | 0.27 | 7.64E-05 |  |  |
|  | rs10919209 | 1 | 169657407 | T | G | SJLIFE1 Survivors | -0.43 | 0.10 | 1.07E-05 |  |  |
|  |  |  |  |  |  | SJLIFE2 Survivors | -0.77 | 0.19 | 6.57E-05 |  |  |
|  |  |  |  |  |  | Combined Survivors | -0.50 | 0.09 | 8.62E-09 | 0.11 | 60.82 |
|  |  |  |  |  |  | Community Controls | -1.08 | 0.27 | 7.93E-05 |  |  |
|  | rs11484813 | 1 | 169659723 | C | T | SJLIFE1 Survivors | -0.43 | 0.10 | 8.90E-06 |  |  |
|  |  |  |  |  |  | SJLIFE2 Survivors | -0.77 | 0.19 | 6.57E-05 |  |  |
|  |  |  |  |  |  | Combined Survivors | -0.50 | 0.09 | 6.95E-09 | 0.11 | 59.97 |
|  |  |  |  |  |  | Community Controls | -1.09 | 0.27 | 6.76E-05 |  |  |
|  | rs11487550 | 1 | 169659921 | A | C | SJLIFE1 Survivors | -0.42 | 0.10 | 1.36E-05 |  |  |
|  |  |  |  |  |  | SJLIFE2 Survivors | -0.78 | 0.19 | 6.15E-05 |  |  |
|  |  |  |  |  |  | Combined Survivors | -0.49 | 0.09 | 1.09E-08 | 0.10 | 62.84 |
|  |  |  |  |  |  | Community Controls | -1.09 | 0.27 | 6.76E-05 |  |  |
|  | rs2420379 | 1 | 169661126 | G | A | SJLIFE1 Survivors | 0.41 | 0.10 | 2.80E-05 |  |  |
|  |  |  |  |  |  | SJLIFE2 Survivors | 0.79 | 0.19 | 2.75E-05 |  |  |
|  |  |  |  |  |  | Combined Survivors | 0.49 | 0.09 | 1.35E-08 | 0.07 | 69.71 |
|  |  |  |  |  |  | Community Controls | 1.07 | 0.27 | 9.56E-05 |  |  |
|  | rs10919211 | 1 | 169661387 | G | A | SJLIFE1 Survivors | -0.43 | 0.10 | 8.89E-06 |  |  |
|  |  |  |  |  |  | SJLIFE2 Survivors | -0.76 | 0.19 | 8.37E-05 |  |  |
|  |  |  |  |  |  | Combined Survivors | -0.50 | 0.09 | 8.16E-09 | 0.13 | 57.06 |
|  |  |  |  |  |  | Community Controls | -1.09 | 0.27 | 6.70E-05 |  |  |
|  | rs10919212 | 1 | 169661694 | G | A | SJLIFE1 Survivors | 0.41 | 0.10 | 2.68E-05 |  |  |
|  |  |  |  |  |  | SJLIFE2 Survivors | 0.78 | 0.19 | 2.87E-05 |  |  |
|  |  |  |  |  |  | Combined Survivors | 0.49 | 0.09 | 1.33E-08 | 0.07 | 69.32 |
|  |  |  |  |  |  | Community Controls | 1.08 | 0.27 | 8.24E-05 |  |  |
|  | rs10919215 | 1 | 169662405 | C | T | SJLIFE1 Survivors | -0.43 | 0.10 | 8.96E-06 |  |  |
|  |  |  |  |  |  | SJLIFE2 Survivors | -0.77 | 0.19 | 6.57E-05 |  |  |
|  |  |  |  |  |  | Combined Survivors | -0.50 | 0.09 | 6.96E-09 | 0.11 | 59.86 |
|  |  |  |  |  |  | Community Controls | -1.08 | 0.27 | 7.93E-05 |  |  |
|  | rs4450069 | 1 | 169663031 | T | A | SJLIFE1 Survivors | 0.41 | 0.10 | 2.02E-05 |  |  |
|  |  |  |  |  |  | SJLIFE2 Survivors | 0.79 | 0.19 | 2.75E-05 |  |  |
|  |  |  |  |  |  | Combined Survivors | 0.49 | 0.09 | 9.29E-09 | 0.07 | 68.75 |
|  |  |  |  |  |  | Community Controls | 1.08 | 0.27 | 8.24E-05 |  |  |
|  | rs4508079 | 1 | 169663572 | A | G | SJLIFE1 Survivors | -0.43 | 0.10 | 9.68E-06 |  |  |
|  |  |  |  |  |  | SJLIFE2 Survivors | -0.77 | 0.19 | 6.06E-05 |  |  |
|  |  |  |  |  |  | Combined Survivors | -0.50 | 0.09 | 7.24E-09 | 0.11 | 61.10 |
|  |  |  |  |  |  | Community Controls | -1.11 | 0.27 | 5.63E-05 |  |  |
|  | rs7530405 | 1 | 169663729 | G | A | SJLIFE1 Survivors | 0.41 | 0.10 | 2.39E-05 |  |  |
|  |  |  |  |  |  | SJLIFE2 Survivors | 0.78 | 0.19 | 2.87E-05 |  |  |
|  |  |  |  |  |  | Combined Survivors | 0.49 | 0.09 | 1.16E-08 | 0.07 | 68.93 |
|  |  |  |  |  |  | Community Controls | 1.08 | 0.27 | 8.24E-05 |  |  |
|  | rs7552947 | 1 | 169663803 | C | T | SJLIFE1 Survivors | -0.42 | 0.10 | 1.35E-05 |  |  |
|  |  |  |  |  |  | SJLIFE2 Survivors | -0.77 | 0.19 | 6.57E-05 |  |  |
|  |  |  |  |  |  | Combined Survivors | -0.49 | 0.09 | 1.11E-08 | 0.11 | 61.59 |
|  |  |  |  |  |  | Community Controls | -1.07 | 0.27 | 1.02E-04 |  |  |
|  | rs10753791 | 1 | 169664606 | G | A | SJLIFE1 Survivors | 0.40 | 0.10 | 3.08E-05 |  |  |
|  |  |  |  |  |  | SJLIFE2 Survivors | 0.79 | 0.19 | 2.75E-05 |  |  |
|  |  |  |  |  |  | Combined Survivors | 0.48 | 0.09 | 1.53E-08 | 0.07 | 70.23 |
|  |  |  |  |  |  | Community Controls | 1.08 | 0.27 | 8.46E-05 |  |  |
|  | rs10919216 | 1 | 169664707 | T | C | SJLIFE1 Survivors | -0.43 | 0.10 | 1.62E-05 |  |  |
|  |  |  |  |  |  | SJLIFE2 Survivors | -0.74 | 0.19 | 1.42E-04 |  |  |
|  |  |  |  |  |  | Combined Survivors | -0.49 | 0.09 | 2.23E-08 | 0.15 | 52.53 |
|  |  |  |  |  |  | Community Controls | -1.16 | 0.28 | 3.28E-05 |  |  |
|  | rs12138071 | 1 | 169665259 | G | A | SJLIFE1 Survivors | 0.41 | 0.10 | 2.63E-05 |  |  |
|  |  |  |  |  |  | SJLIFE2 Survivors | 0.74 | 0.19 | 7.65E-05 |  |  |
|  |  |  |  |  |  | Combined Survivors | 0.48 | 0.09 | 2.46E-08 | 0.11 | 61.00 |
|  |  |  |  |  |  | Community Controls | 1.07 | 0.27 | 9.56E-05 |  |  |
|  | rs7517544 | 1 | 169666999 | C | T | SJLIFE1 Survivors | -0.43 | 0.10 | 8.18E-06 |  |  |
|  |  |  |  |  |  | SJLIFE2 Survivors | -0.78 | 0.19 | 6.15E-05 |  |  |
|  |  |  |  |  |  | Combined Survivors | -0.50 | 0.09 | 6.10E-09 | 0.11 | 60.39 |
|  |  |  |  |  |  | Community Controls | -1.11 | 0.27 | 5.65E-05 |  |  |
|  | rs12116948 | 1 | 169668087 | C | T | SJLIFE1 Survivors | -0.42 | 0.10 | 1.65E-05 |  |  |
|  |  |  |  |  |  | SJLIFE2 Survivors | -0.73 | 0.19 | 1.89E-04 |  |  |
|  |  |  |  |  |  | Combined Survivors | -0.49 | 0.09 | 2.77E-08 | 0.16 | 48.32 |
|  |  |  |  |  |  | Community Controls | -1.13 | 0.27 | 4.88E-05 |  |  |
|  | rs7415448 | 1 | 169669121 | C | T | SJLIFE1 Survivors | 0.39 | 0.10 | 4.70E-05 |  |  |
|  |  |  |  |  |  | SJLIFE2 Survivors | 0.78 | 0.19 | 2.87E-05 |  |  |
|  |  |  |  |  |  | Combined Survivors | 0.48 | 0.09 | 2.58E-08 | 0.06 | 71.18 |
|  |  |  |  |  |  | Community Controls | 1.07 | 0.27 | 1.05E-04 |  |  |
|  | rs12086410 | 1 | 169669160 | T | C | SJLIFE1 Survivors | -0.43 | 0.10 | 1.06E-05 |  |  |
|  |  |  |  |  |  | SJLIFE2 Survivors | -0.77 | 0.19 | 6.77E-05 |  |  |
|  |  |  |  |  |  | Combined Survivors | -0.50 | 0.09 | 8.75E-09 | 0.11 | 60.81 |
|  |  |  |  |  |  | Community Controls | -1.11 | 0.27 | 5.48E-05 |  |  |
|  | rs7413295 | 1 | 169669196 | G | T | SJLIFE1 Survivors | -0.42 | 0.10 | 1.60E-05 |  |  |
|  |  |  |  |  |  | SJLIFE2 Survivors | -0.78 | 0.19 | 6.23E-05 |  |  |
|  |  |  |  |  |  | Combined Survivors | -0.49 | 0.09 | 1.35E-08 | 0.10 | 63.72 |
|  |  |  |  |  |  | Community Controls | -1.12 | 0.27 | 4.87E-05 |  |  |
|  | rs12086443 | 1 | 169669232 | T | C | SJLIFE1 Survivors | -0.42 | 0.10 | 1.80E-05 |  |  |
|  |  |  |  |  |  | SJLIFE2 Survivors | -0.76 | 0.19 | 8.27E-05 |  |  |
|  |  |  |  |  |  | Combined Survivors | -0.49 | 0.09 | 1.81E-08 | 0.11 | 60.78 |
|  |  |  |  |  |  | Community Controls | -1.19 | 0.28 | 3.03E-05 |  |  |
|  | rs7513508 | 1 | 169670762 | G | A | SJLIFE1 Survivors | -0.43 | 0.10 | 1.21E-05 |  |  |
|  |  |  |  |  |  | SJLIFE2 Survivors | -0.77 | 0.19 | 6.13E-05 |  |  |
|  |  |  |  |  |  | Combined Survivors | -0.50 | 0.09 | 9.44E-09 | 0.10 | 62.09 |
|  |  |  |  |  |  | Community Controls | -1.11 | 0.27 | 4.67E-05 |  |  |
|  | rs11322364 | 1 | 169670798 | G | GT | SJLIFE1 Survivors | 0.40 | 0.10 | 4.05E-05 |  |  |
|  |  |  |  |  |  | SJLIFE2 Survivors | 0.79 | 0.19 | 2.72E-05 |  |  |
|  |  |  |  |  |  | Combined Survivors | 0.48 | 0.09 | 2.07E-08 | 0.06 | 70.93 |
|  |  |  |  |  |  | Community Controls | 1.12 | 0.27 | 4.92E-05 |  |  |
|  | rs1963141 | 1 | 169672590 | A | G | SJLIFE1 Survivors | -0.42 | 0.10 | 1.64E-05 |  |  |
|  |  |  |  |  |  | SJLIFE2 Survivors | -0.77 | 0.19 | 5.87E-05 |  |  |
|  |  |  |  |  |  | Combined Survivors | -0.49 | 0.09 | 1.31E-08 | 0.10 | 63.96 |
|  |  |  |  |  |  | Community Controls | -1.12 | 0.27 | 3.93E-05 |  |  |
|  | rs7544874 | 1 | 169673308 | A | G | SJLIFE1 Survivors | -0.42 | 0.10 | 1.71E-05 |  |  |
|  |  |  |  |  |  | SJLIFE2 Survivors | -0.78 | 0.19 | 4.66E-05 |  |  |
|  |  |  |  |  |  | Combined Survivors | -0.49 | 0.09 | 1.18E-08 | 0.09 | 66.06 |
|  |  |  |  |  |  | Community Controls | -1.14 | 0.27 | 3.09E-05 |  |  |
|  | rs11407937 | 1 | 169673394 | - | A | SJLIFE1 Survivors | -0.41 | 0.10 | 2.63E-05 |  |  |
|  |  |  |  |  |  | SJLIFE2 Survivors | -0.78 | 0.19 | 5.30E-05 |  |  |
|  |  |  |  |  |  | Combined Survivors | -0.48 | 0.09 | 2.16E-08 | 0.08 | 67.00 |
|  |  |  |  |  |  | Community Controls | -1.14 | 0.27 | 3.17E-05 |  |  |
|  | rs7537814 | 1 | 169674563 | T | A | SJLIFE1 Survivors | -0.44 | 0.10 | 3.89E-06 |  |  |
|  |  |  |  |  |  | SJLIFE2 Survivors | -0.79 | 0.19 | 4.85E-05 |  |  |
|  |  |  |  |  |  | Combined Survivors | -0.51 | 0.09 | 2.41E-09 | 0.10 | 61.96 |
|  |  |  |  |  |  | Community Controls | -1.09 | 0.27 | 6.86E-05 |  |  |
|  | rs7525857 | 1 | 169674567 | C | T | SJLIFE1 Survivors | -0.44 | 0.10 | 3.89E-06 |  |  |
|  |  |  |  |  |  | SJLIFE2 Survivors | -0.79 | 0.19 | 4.85E-05 |  |  |
|  |  |  |  |  |  | Combined Survivors | -0.51 | 0.09 | 2.41E-09 | 0.10 | 61.96 |
|  |  |  |  |  |  | Community Controls | -1.09 | 0.27 | 6.86E-05 |  |  |
|  | rs6690763 | 1 | 169676313 | T | A | SJLIFE1 Survivors | -0.42 | 0.10 | 1.55E-05 |  |  |
|  |  |  |  |  |  | SJLIFE2 Survivors | -0.78 | 0.19 | 4.66E-05 |  |  |
|  |  |  |  |  |  | Combined Survivors | -0.49 | 0.09 | 1.05E-08 | 0.09 | 65.71 |
|  |  |  |  |  |  | Community Controls | -1.13 | 0.27 | 3.51E-05 |  |  |
|  | rs6678930 | 1 | 169677023 | G | C | SJLIFE1 Survivors | -0.42 | 0.10 | 1.63E-05 |  |  |
|  |  |  |  |  |  | SJLIFE2 Survivors | -0.78 | 0.19 | 4.69E-05 |  |  |
|  |  |  |  |  |  | Combined Survivors | -0.49 | 0.09 | 1.13E-08 | 0.09 | 65.88 |
|  |  |  |  |  |  | Community Controls | -1.11 | 0.27 | 4.37E-05 |  |  |
|  | rs1011267 | 1 | 169677720 | A | G | SJLIFE1 Survivors | -0.43 | 0.10 | 9.55E-06 |  |  |
|  |  |  |  |  |  | SJLIFE2 Survivors | -0.78 | 0.19 | 4.85E-05 |  |  |
|  |  |  |  |  |  | Combined Survivors | -0.50 | 0.09 | 6.30E-09 | 0.10 | 63.83 |
|  |  |  |  |  |  | Community Controls | -1.12 | 0.27 | 4.05E-05 |  |  |
| EAA-Hannum | rs28752872 | 6 | 31334973 | A | G | SJLIFE1 Survivors | 0.54 | 0.14 | 1.09E-04 |  |  |
|  |  |  |  |  |  | SJLIFE2 Survivors | 0.93 | 0.22 | 3.57E-05 |  |  |
|  |  |  |  |  |  | Combined Survivors | 0.65 | 0.12 | 3.88E-08 | 0.14 | 55.10 |
|  |  |  |  |  |  | Community Controls | 0.61 | 0.30 | 4.62E-02 |  |  |
|  | rs28752873 | 6 | 31334975 | T | C | SJLIFE1 Survivors | 0.54 | 0.14 | 1.10E-04 |  |  |
|  |  |  |  |  |  | SJLIFE2 Survivors | 0.93 | 0.22 | 3.57E-05 |  |  |
|  |  |  |  |  |  | Combined Survivors | 0.65 | 0.12 | 3.93E-08 | 0.14 | 55.14 |
|  |  |  |  |  |  | Community Controls | 0.57 | 0.30 | 5.80E-02 |  |  |
|  | rs28752874 | 6 | 31334978 | C | T | SJLIFE1 Survivors | 0.54 | 0.14 | 9.77E-05 |  |  |
|  |  |  |  |  |  | SJLIFE2 Survivors | 0.93 | 0.22 | 3.57E-05 |  |  |
|  |  |  |  |  |  | Combined Survivors | 0.65 | 0.12 | 3.40E-08 | 0.14 | 54.22 |
|  |  |  |  |  |  | Community Controls | 0.61 | 0.30 | 4.59E-02 |  |  |
|  | rs28771423 | 6 | 31347372 | G | A | SJLIFE1 Survivors | 0.58 | 0.14 | 2.37E-05 |  |  |
|  |  |  |  |  |  | SJLIFE2 Survivors | 0.88 | 0.22 | 8.05E-05 |  |  |
|  |  |  |  |  |  | Combined Survivors | 0.67 | 0.12 | 1.23E-08 | 0.25 | 24.68 |
|  |  |  |  |  |  | Community Controls | 0.57 | 0.31 | 6.48E-02 |  |  |
|  | rs112261624 | 6 | 31358410-31358413 | TGT | TGT (dup) | SJLIFE1 Survivors | 0.99 | 0.20 | 8.11E-07 |  |  |
|  |  |  |  |  |  | SJLIFE2 Survivors | 0.82 | 0.32 | 1.02E-02 |  |  |
|  |  |  |  |  |  | Combined Survivors | 0.94 | 0.17 | 2.67E-08 | 0.65 | 0.00 |
|  |  |  |  |  |  | Community Controls | 0.53 | 0.43 | 2.15E-01 |  |  |
|  | rs56189237 | 6 | 31360741 | G | A | SJLIFE1 Survivors | 1.01 | 0.20 | 5.91E-07 |  |  |
|  |  |  |  |  |  | SJLIFE2 Survivors | 0.90 | 0.32 | 5.13E-03 |  |  |
|  |  |  |  |  |  | Combined Survivors | 0.98 | 0.17 | 9.55E-09 | 0.78 | 0.00 |
|  |  |  |  |  |  | Community Controls | 0.46 | 0.43 | 2.86E-01 |  |  |
|  | rs9266634 | 6 | 31379201 | T | A | SJLIFE1 Survivors | -0.67 | 0.14 | 1.51E-06 |  |  |
|  |  |  |  |  |  | SJLIFE2 Survivors | -0.77 | 0.23 | 1.03E-03 |  |  |
|  |  |  |  |  |  | Combined Survivors | -0.69 | 0.12 | 5.41E-09 | 0.71 | 0.00 |
|  |  |  |  |  |  | Community Controls | -0.64 | 0.30 | 3.07E-02 |  |  |
|  | rs2523465 | 6 | 31395313 | G | C | SJLIFE1 Survivors | 0.82 | 0.15 | 1.11E-07 |  |  |
|  |  |  |  |  |  | SJLIFE2 Survivors | 0.58 | 0.25 | 1.99E-02 |  |  |
|  |  |  |  |  |  | Combined Survivors | 0.75 | 0.13 | 8.49E-09 | 0.41 | 0.00 |
|  |  |  |  |  |  | Community Controls | -0.07 | 0.34 | 8.35E-01 |  |  |
|  | rs2523463 | 6 | 31395724 | A | C | SJLIFE1 Survivors | 0.75 | 0.15 | 1.24E-06 |  |  |
|  |  |  |  |  |  | SJLIFE2 Survivors | 0.67 | 0.24 | 6.05E-03 |  |  |
|  |  |  |  |  |  | Combined Survivors | 0.73 | 0.13 | 2.32E-08 | 0.79 | 0.00 |
|  |  |  |  |  |  | Community Controls | NA | NA | NA |  |  |
|  | rs2596483 | 6 | 31395903 | A | G | SJLIFE1 Survivors | 0.77 | 0.15 | 2.74E-07 |  |  |
|  |  |  |  |  |  | SJLIFE2 Survivors | 0.67 | 0.24 | 5.35E-03 |  |  |
|  |  |  |  |  |  | Combined Survivors | 0.74 | 0.13 | 4.65E-09 | 0.75 | 0.00 |
|  |  |  |  |  |  | Community Controls | -0.05 | 0.33 | 8.91E-01 |  |  |

Abbreviations: GWAS, genome-wide association study; EAA, epigenetic age acceleration; Chr, chromosome; SE, standard error; SJLIFE1 Survivors, the first discovery data set of 2138 survivors included in our previously published study (ref. 6); SJLIFE2 Survivors, the second discovery data set of 502 children and adolescent survivors; Combined Survivors, a combined set (meta-GWAS analysis) of the two discovery data sets (SJLIFE1 and SJLIFE2); Community Controls, a set of 282 community controls.

**Table S2. SNPs significantly associated with IEAA-Horvath and EEAA-Hannum (in meta-GWAS analysis) among survivors (SJLIFE1, SJLIFE2) and controls.**

| GWAS | SNP | Chr | Pos_hg38 | Effect allele | Other allele | Population | Effect size | (SE) | *P* | *P*_het_ | I^2^ (%) |
| --- | --- | --- | --- | --- | --- | --- | --- | --- | --- | --- | --- |
| IEAA-Horvath | rs3917698 | 1 | 169618879 | G | A | SJLIFE1 Survivors | -0.43 | 0.10 | 7.88E-06 |  |  |
|  |  |  |  |  |  | SJLIFE2 Survivors | -0.83 | 0.19 | 1.35E-05 |  |  |
|  |  |  |  |  |  | Combined Survivors | -0.51 | 0.08 | 2.20E-09 | 0.06 | 72.44 |
|  |  |  |  |  |  | Community Controls | -1.03 | 0.27 | 1.67E-04 |  |  |
|  | rs3917688 | 1 | 169621842 | C | T | SJLIFE1 Survivors | -0.45 | 0.09 | 1.46E-06 |  |  |
|  |  |  |  |  |  | SJLIFE2 Survivors | -0.81 | 0.19 | 2.39E-05 |  |  |
|  |  |  |  |  |  | Combined Survivors | -0.53 | 0.08 | 4.96E-10 | 0.09 | 64.85 |
|  |  |  |  |  |  | Community Controls | -1.08 | 0.27 | 8.35E-05 |  |  |
|  | rs3917683 | 1 | 169622135 | T | C | SJLIFE1 Survivors | -0.42 | 0.10 | 8.90E-06 |  |  |
|  |  |  |  |  |  | SJLIFE2 Survivors | -0.83 | 0.19 | 1.36E-05 |  |  |
|  |  |  |  |  |  | Combined Survivors | -0.51 | 0.08 | 2.56E-09 | 0.06 | 72.71 |
|  |  |  |  |  |  | Community Controls | -1.02 | 0.27 | 2.25E-04 |  |  |
|  | rs3917679 | 1 | 169622615 | C | T | SJLIFE1 Survivors | -0.47 | 0.09 | 5.33E-07 |  |  |
|  |  |  |  |  |  | SJLIFE2 Survivors | -0.80 | 0.19 | 3.37E-05 |  |  |
|  |  |  |  |  |  | Combined Survivors | -0.54 | 0.08 | 1.98E-10 | 0.13 | 57.39 |
|  |  |  |  |  |  | Community Controls | -1.08 | 0.27 | 8.41E-05 |  |  |
|  | rs3917672 | 1 | 169623743 | A | G | SJLIFE1 Survivors | -0.46 | 0.09 | 1.01E-06 |  |  |
|  |  |  |  |  |  | SJLIFE2 Survivors | -0.80 | 0.19 | 3.56E-05 |  |  |
|  |  |  |  |  |  | Combined Survivors | -0.53 | 0.08 | 4.15E-10 | 0.12 | 59.05 |
|  |  |  |  |  |  | Community Controls | -1.16 | 0.27 | 2.15E-05 |  |  |
|  | rs2236867 | 1 | 169623749 | A | C | SJLIFE1 Survivors | -0.41 | 0.10 | 1.58E-05 |  |  |
|  |  |  |  |  |  | SJLIFE2 Survivors | -0.82 | 0.19 | 1.44E-05 |  |  |
|  |  |  |  |  |  | Combined Survivors | -0.50 | 0.09 | 5.07E-09 | 0.05 | 73.54 |
|  |  |  |  |  |  | Community Controls | -1.10 | 0.27 | 6.68E-05 |  |  |
|  | rs2236866 | 1 | 169627075 | A | T | SJLIFE1 Survivors | -0.44 | 0.10 | 5.45E-06 |  |  |
|  |  |  |  |  |  | SJLIFE2 Survivors | -0.83 | 0.19 | 1.25E-05 |  |  |
|  |  |  |  |  |  | Combined Survivors | -0.52 | 0.09 | 1.35E-09 | 0.06 | 71.71 |
|  |  |  |  |  |  | Community Controls | -1.04 | 0.27 | 1.43E-04 |  |  |
|  | rs764199 | 1 | 169628000 | T | A | SJLIFE1 Survivors | -0.46 | 0.10 | 1.83E-06 |  |  |
|  |  |  |  |  |  | SJLIFE2 Survivors | -0.84 | 0.19 | 9.05E-06 |  |  |
|  |  |  |  |  |  | Combined Survivors | -0.54 | 0.09 | 3.01E-10 | 0.07 | 70.27 |
|  |  |  |  |  |  | Community Controls | -1.05 | 0.27 | 1.52E-04 |  |  |
|  | rs732314 | 1 | 169630016 | T | C | SJLIFE1 Survivors | -0.50 | 0.09 | 9.46E-08 |  |  |
|  |  |  |  |  |  | SJLIFE2 Survivors | -0.80 | 0.19 | 3.29E-05 |  |  |
|  |  |  |  |  |  | Combined Survivors | -0.56 | 0.08 | 2.79E-11 | 0.16 | 48.21 |
|  |  |  |  |  |  | Community Controls | -1.05 | 0.27 | 1.17E-04 |  |  |
|  | rs3917647 | 1 | 169632102 | G | A | SJLIFE1 Survivors | -0.47 | 0.09 | 6.31E-07 |  |  |
|  |  |  |  |  |  | SJLIFE2 Survivors | -0.78 | 0.19 | 4.58E-05 |  |  |
|  |  |  |  |  |  | Combined Survivors | -0.53 | 0.08 | 2.91E-10 | 0.14 | 53.48 |
|  |  |  |  |  |  | Community Controls | -1.02 | 0.27 | 1.80E-04 |  |  |
|  | rs1800807 | 1 | 169632197 | C | G | SJLIFE1 Survivors | -0.44 | 0.10 | 4.34E-06 |  |  |
|  |  |  |  |  |  | SJLIFE2 Survivors | -0.84 | 0.19 | 1.10E-05 |  |  |
|  |  |  |  |  |  | Combined Survivors | -0.52 | 0.09 | 9.40E-10 | 0.06 | 71.66 |
|  |  |  |  |  |  | Community Controls | -0.97 | 0.27 | 4.20E-04 |  |  |
|  | rs6666554 | 1 | 169632871 | A | G | SJLIFE1 Survivors | -0.45 | 0.10 | 2.24E-06 |  |  |
|  |  |  |  |  |  | SJLIFE2 Survivors | -0.79 | 0.19 | 2.89E-05 |  |  |
|  |  |  |  |  |  | Combined Survivors | -0.52 | 0.08 | 8.23E-10 | 0.11 | 61.92 |
|  |  |  |  |  |  | Community Controls | -0.98 | 0.27 | 3.74E-04 |  |  |
|  | rs2205892 | 1 | 169635516 | G | A | SJLIFE1 Survivors | -0.45 | 0.10 | 2.97E-06 |  |  |
|  |  |  |  |  |  | SJLIFE2 Survivors | -0.78 | 0.19 | 3.63E-05 |  |  |
|  |  |  |  |  |  | Combined Survivors | -0.52 | 0.08 | 1.30E-09 | 0.11 | 60.28 |
|  |  |  |  |  |  | Community Controls | -1.02 | 0.27 | 2.28E-04 |  |  |
|  | rs10800460 | 1 | 169636770 | T | C | SJLIFE1 Survivors | -0.44 | 0.10 | 4.11E-06 |  |  |
|  |  |  |  |  |  | SJLIFE2 Survivors | -0.79 | 0.19 | 3.22E-05 |  |  |
|  |  |  |  |  |  | Combined Survivors | -0.51 | 0.09 | 1.75E-09 | 0.10 | 63.04 |
|  |  |  |  |  |  | Community Controls | -0.99 | 0.27 | 3.22E-04 |  |  |
|  | rs10753788 | 1 | 169637035 | A | G | SJLIFE1 Survivors | -0.43 | 0.10 | 1.01E-05 |  |  |
|  |  |  |  |  |  | SJLIFE2 Survivors | -0.81 | 0.19 | 2.57E-05 |  |  |
|  |  |  |  |  |  | Combined Survivors | -0.51 | 0.09 | 4.33E-09 | 0.07 | 68.67 |
|  |  |  |  |  |  | Community Controls | -0.99 | 0.29 | 6.15E-04 |  |  |
|  | rs9659257 | 1 | 169638830 | C | G | SJLIFE1 Survivors | -0.45 | 0.10 | 2.59E-06 |  |  |
|  |  |  |  |  |  | SJLIFE2 Survivors | -0.78 | 0.19 | 3.49E-05 |  |  |
|  |  |  |  |  |  | Combined Survivors | -0.52 | 0.08 | 1.10E-09 | 0.11 | 60.52 |
|  |  |  |  |  |  | Community Controls | -1.01 | 0.27 | 2.37E-04 |  |  |
|  | rs6427205 | 1 | 169642234 | T | G | SJLIFE1 Survivors | -0.46 | 0.10 | 1.67E-06 |  |  |
|  |  |  |  |  |  | SJLIFE2 Survivors | -0.78 | 0.19 | 3.48E-05 |  |  |
|  |  |  |  |  |  | Combined Survivors | -0.52 | 0.08 | 6.62E-10 | 0.12 | 58.58 |
|  |  |  |  |  |  | Community Controls | -1.01 | 0.27 | 2.58E-04 |  |  |
|  | rs10800462 | 1 | 169646782 | A | G | SJLIFE1 Survivors | -0.40 | 0.10 | 2.50E-05 |  |  |
|  |  |  |  |  |  | SJLIFE2 Survivors | -0.78 | 0.19 | 3.26E-05 |  |  |
|  |  |  |  |  |  | Combined Survivors | -0.48 | 0.08 | 1.39E-08 | 0.07 | 69.11 |
|  |  |  |  |  |  | Community Controls | -1.07 | 0.27 | 1.10E-04 |  |  |
|  | rs7551819 | 1 | 169647471 | A | C | SJLIFE1 Survivors | -0.39 | 0.09 | 3.41E-05 |  |  |
|  |  |  |  |  |  | SJLIFE2 Survivors | -0.78 | 0.19 | 3.26E-05 |  |  |
|  |  |  |  |  |  | Combined Survivors | -0.47 | 0.08 | 2.04E-08 | 0.07 | 70.39 |
|  |  |  |  |  |  | Community Controls | -1.06 | 0.27 | 1.28E-04 |  |  |
|  | rs2142759 | 1 | 169649582 | A | G | SJLIFE1 Survivors | -0.42 | 0.09 | 1.09E-05 |  |  |
|  |  |  |  |  |  | SJLIFE2 Survivors | -0.78 | 0.19 | 3.26E-05 |  |  |
|  |  |  |  |  |  | Combined Survivors | -0.49 | 0.08 | 5.30E-09 | 0.09 | 66.22 |
|  |  |  |  |  |  | Community Controls | -1.06 | 0.27 | 1.28E-04 |  |  |
|  | rs6687517 | 1 | 169652823 | C | T | SJLIFE1 Survivors | 0.44 | 0.10 | 4.43E-06 |  |  |
|  |  |  |  |  |  | SJLIFE2 Survivors | 0.76 | 0.19 | 9.01E-05 |  |  |
|  |  |  |  |  |  | Combined Survivors | 0.50 | 0.09 | 4.13E-09 | 0.14 | 54.80 |
|  |  |  |  |  |  | Community Controls | 1.09 | 0.27 | 9.30E-05 |  |  |
|  | rs10800463 | 1 | 169653866 | G | A | SJLIFE1 Survivors | 0.44 | 0.10 | 3.76E-06 |  |  |
|  |  |  |  |  |  | SJLIFE2 Survivors | 0.75 | 0.19 | 9.48E-05 |  |  |
|  |  |  |  |  |  | Combined Survivors | 0.50 | 0.09 | 3.50E-09 | 0.15 | 52.58 |
|  |  |  |  |  |  | Community Controls | 1.06 | 0.27 | 1.42E-04 |  |  |
|  | rs1569473 | 1 | 169654788 | G | A | SJLIFE1 Survivors | -0.40 | 0.10 | 2.23E-05 |  |  |
|  |  |  |  |  |  | SJLIFE2 Survivors | -0.78 | 0.19 | 3.03E-05 |  |  |
|  |  |  |  |  |  | Combined Survivors | -0.48 | 0.08 | 1.18E-08 | 0.07 | 69.50 |
|  |  |  |  |  |  | Community Controls | -1.08 | 0.28 | 1.15E-04 |  |  |
|  | rs2179188 | 1 | 169654939 | G | A | SJLIFE1 Survivors | 0.44 | 0.10 | 3.68E-06 |  |  |
|  |  |  |  |  |  | SJLIFE2 Survivors | 0.75 | 0.19 | 9.19E-05 |  |  |
|  |  |  |  |  |  | Combined Survivors | 0.50 | 0.09 | 3.35E-09 | 0.14 | 52.99 |
|  |  |  |  |  |  | Community Controls | 1.04 | 0.27 | 1.77E-04 |  |  |
|  | rs10919209 | 1 | 169657407 | G | T | SJLIFE1 Survivors | 0.43 | 0.10 | 6.20E-06 |  |  |
|  |  |  |  |  |  | SJLIFE2 Survivors | 0.75 | 0.19 | 9.19E-05 |  |  |
|  |  |  |  |  |  | Combined Survivors | 0.50 | 0.09 | 6.08E-09 | 0.13 | 56.06 |
|  |  |  |  |  |  | Community Controls | 1.05 | 0.27 | 1.78E-04 |  |  |
|  | rs11484813 | 1 | 169659723 | T | C | SJLIFE1 Survivors | 0.43 | 0.10 | 5.23E-06 |  |  |
|  |  |  |  |  |  | SJLIFE2 Survivors | 0.75 | 0.19 | 9.19E-05 |  |  |
|  |  |  |  |  |  | Combined Survivors | 0.50 | 0.09 | 5.03E-09 | 0.14 | 55.22 |
|  |  |  |  |  |  | Community Controls | 1.06 | 0.27 | 1.52E-04 |  |  |
|  | rs11487550 | 1 | 169659921 | C | A | SJLIFE1 Survivors | 0.43 | 0.10 | 7.91E-06 |  |  |
|  |  |  |  |  |  | SJLIFE2 Survivors | 0.76 | 0.19 | 9.16E-05 |  |  |
|  |  |  |  |  |  | Combined Survivors | 0.49 | 0.09 | 8.07E-09 | 0.12 | 57.73 |
|  |  |  |  |  |  | Community Controls | 1.06 | 0.27 | 1.52E-04 |  |  |
|  | rs2420379 | 1 | 169661126 | A | G | SJLIFE1 Survivors | -0.40 | 0.10 | 2.51E-05 |  |  |
|  |  |  |  |  |  | SJLIFE2 Survivors | -0.78 | 0.19 | 3.03E-05 |  |  |
|  |  |  |  |  |  | Combined Survivors | -0.48 | 0.08 | 1.34E-08 | 0.07 | 69.67 |
|  |  |  |  |  |  | Community Controls | -1.05 | 0.27 | 1.57E-04 |  |  |
|  | rs10919211 | 1 | 169661387 | A | G | SJLIFE1 Survivors | 0.44 | 0.10 | 4.87E-06 |  |  |
|  |  |  |  |  |  | SJLIFE2 Survivors | 0.75 | 0.19 | 1.06E-04 |  |  |
|  |  |  |  |  |  | Combined Survivors | 0.50 | 0.09 | 5.07E-09 | 0.15 | 52.66 |
|  |  |  |  |  |  | Community Controls | 1.05 | 0.27 | 1.55E-04 |  |  |
|  | rs10919212 | 1 | 169661694 | A | G | SJLIFE1 Survivors | -0.40 | 0.10 | 2.56E-05 |  |  |
|  |  |  |  |  |  | SJLIFE2 Survivors | -0.78 | 0.19 | 3.01E-05 |  |  |
|  |  |  |  |  |  | Combined Survivors | -0.48 | 0.08 | 1.37E-08 | 0.07 | 69.85 |
|  |  |  |  |  |  | Community Controls | -1.06 | 0.27 | 1.36E-04 |  |  |
|  | rs10919215 | 1 | 169662405 | T | C | SJLIFE1 Survivors | 0.44 | 0.10 | 4.81E-06 |  |  |
|  |  |  |  |  |  | SJLIFE2 Survivors | 0.75 | 0.19 | 9.19E-05 |  |  |
|  |  |  |  |  |  | Combined Survivors | 0.50 | 0.09 | 4.54E-09 | 0.14 | 54.54 |
|  |  |  |  |  |  | Community Controls | 1.05 | 0.27 | 1.78E-04 |  |  |
|  | rs4450069 | 1 | 169663031 | A | T | SJLIFE1 Survivors | -0.41 | 0.10 | 2.03E-05 |  |  |
|  |  |  |  |  |  | SJLIFE2 Survivors | -0.78 | 0.19 | 3.03E-05 |  |  |
|  |  |  |  |  |  | Combined Survivors | -0.48 | 0.08 | 1.05E-08 | 0.07 | 69.14 |
|  |  |  |  |  |  | Community Controls | -1.06 | 0.27 | 1.36E-04 |  |  |
|  | rs4508079 | 1 | 169663572 | G | A | SJLIFE1 Survivors | 0.43 | 0.10 | 5.93E-06 |  |  |
|  |  |  |  |  |  | SJLIFE2 Survivors | 0.76 | 0.19 | 9.01E-05 |  |  |
|  |  |  |  |  |  | Combined Survivors | 0.50 | 0.09 | 5.71E-09 | 0.13 | 56.06 |
|  |  |  |  |  |  | Community Controls | 1.07 | 0.28 | 1.35E-04 |  |  |
|  | rs7530405 | 1 | 169663729 | A | G | SJLIFE1 Survivors | -0.40 | 0.10 | 2.33E-05 |  |  |
|  |  |  |  |  |  | SJLIFE2 Survivors | -0.78 | 0.19 | 3.01E-05 |  |  |
|  |  |  |  |  |  | Combined Survivors | -0.48 | 0.08 | 1.23E-08 | 0.07 | 69.55 |
|  |  |  |  |  |  | Community Controls | -1.06 | 0.27 | 1.36E-04 |  |  |
|  | rs7552947 | 1 | 169663803 | T | C | SJLIFE1 Survivors | 0.43 | 0.10 | 7.68E-06 |  |  |
|  |  |  |  |  |  | SJLIFE2 Survivors | 0.75 | 0.19 | 9.19E-05 |  |  |
|  |  |  |  |  |  | Combined Survivors | 0.49 | 0.09 | 7.69E-09 | 0.13 | 56.85 |
|  |  |  |  |  |  | Community Controls | 1.06 | 0.28 | 1.50E-04 |  |  |
|  | rs10753791 | 1 | 169664606 | A | G | SJLIFE1 Survivors | -0.40 | 0.10 | 2.80E-05 |  |  |
|  |  |  |  |  |  | SJLIFE2 Survivors | -0.78 | 0.19 | 3.03E-05 |  |  |
|  |  |  |  |  |  | Combined Survivors | -0.48 | 0.08 | 1.54E-08 | 0.07 | 70.22 |
|  |  |  |  |  |  | Community Controls | -1.07 | 0.28 | 1.35E-04 |  |  |
|  | rs10919216 | 1 | 169664707 | C | T | SJLIFE1 Survivors | 0.44 | 0.10 | 6.17E-06 |  |  |
|  |  |  |  |  |  | SJLIFE2 Survivors | 0.72 | 0.19 | 1.94E-04 |  |  |
|  |  |  |  |  |  | Combined Survivors | 0.50 | 0.09 | 9.68E-09 | 0.19 | 42.63 |
|  |  |  |  |  |  | Community Controls | 1.12 | 0.28 | 7.92E-05 |  |  |
|  | rs12138071 | 1 | 169665259 | A | G | SJLIFE1 Survivors | -0.40 | 0.10 | 2.93E-05 |  |  |
|  |  |  |  |  |  | SJLIFE2 Survivors | -0.74 | 0.19 | 7.54E-05 |  |  |
|  |  |  |  |  |  | Combined Survivors | -0.47 | 0.08 | 2.89E-08 | 0.10 | 63.01 |
|  |  |  |  |  |  | Community Controls | -1.05 | 0.27 | 1.57E-04 |  |  |
|  | rs7517544 | 1 | 169666999 | T | C | SJLIFE1 Survivors | 0.44 | 0.10 | 4.96E-06 |  |  |
|  |  |  |  |  |  | SJLIFE2 Survivors | 0.76 | 0.19 | 9.16E-05 |  |  |
|  |  |  |  |  |  | Combined Survivors | 0.50 | 0.09 | 4.74E-09 | 0.14 | 55.15 |
|  |  |  |  |  |  | Community Controls | 1.07 | 0.28 | 1.37E-04 |  |  |
|  | rs12116948 | 1 | 169668087 | T | C | SJLIFE1 Survivors | 0.44 | 0.10 | 6.43E-06 |  |  |
|  |  |  |  |  |  | SJLIFE2 Survivors | 0.72 | 0.19 | 2.20E-04 |  |  |
|  |  |  |  |  |  | Combined Survivors | 0.49 | 0.09 | 1.11E-08 | 0.20 | 40.39 |
|  |  |  |  |  |  | Community Controls | 1.08 | 0.28 | 1.19E-04 |  |  |
|  | rs7415448 | 1 | 169669121 | T | C | SJLIFE1 Survivors | -0.39 | 0.10 | 4.28E-05 |  |  |
|  |  |  |  |  |  | SJLIFE2 Survivors | -0.78 | 0.19 | 3.01E-05 |  |  |
|  |  |  |  |  |  | Combined Survivors | -0.47 | 0.08 | 2.50E-08 | 0.06 | 71.47 |
|  |  |  |  |  |  | Community Controls | -1.06 | 0.28 | 1.54E-04 |  |  |
|  | rs12086410 | 1 | 169669160 | C | T | SJLIFE1 Survivors | 0.43 | 0.10 | 6.40E-06 |  |  |
|  |  |  |  |  |  | SJLIFE2 Survivors | 0.76 | 0.19 | 9.61E-05 |  |  |
|  |  |  |  |  |  | Combined Survivors | 0.50 | 0.09 | 6.60E-09 | 0.13 | 56.23 |
|  |  |  |  |  |  | Community Controls | 1.08 | 0.28 | 1.18E-04 |  |  |
|  | rs7413295 | 1 | 169669196 | T | G | SJLIFE1 Survivors | 0.43 | 0.10 | 7.99E-06 |  |  |
|  |  |  |  |  |  | SJLIFE2 Survivors | 0.76 | 0.19 | 9.48E-05 |  |  |
|  |  |  |  |  |  | Combined Survivors | 0.49 | 0.09 | 8.45E-09 | 0.12 | 57.87 |
|  |  |  |  |  |  | Community Controls | 1.08 | 0.28 | 1.10E-04 |  |  |
|  | rs12086443 | 1 | 169669232 | C | T | SJLIFE1 Survivors | 0.42 | 0.10 | 1.01E-05 |  |  |
|  |  |  |  |  |  | SJLIFE2 Survivors | 0.75 | 0.19 | 1.08E-04 |  |  |
|  |  |  |  |  |  | Combined Survivors | 0.49 | 0.09 | 1.17E-08 | 0.13 | 56.54 |
|  |  |  |  |  |  | Community Controls | 1.16 | 0.28 | 5.84E-05 |  |  |
|  | rs7513508 | 1 | 169670762 | A | G | SJLIFE1 Survivors | 0.43 | 0.10 | 7.75E-06 |  |  |
|  |  |  |  |  |  | SJLIFE2 Survivors | 0.76 | 0.19 | 8.43E-05 |  |  |
|  |  |  |  |  |  | Combined Survivors | 0.49 | 0.09 | 7.43E-09 | 0.12 | 58.46 |
|  |  |  |  |  |  | Community Controls | 1.08 | 0.27 | 1.04E-04 |  |  |
|  | rs11322364 | 1 | 169670798 | T | - | SJLIFE1 Survivors | -0.39 | 0.10 | 3.94E-05 |  |  |
|  |  |  |  |  |  | SJLIFE2 Survivors | -0.78 | 0.19 | 2.82E-05 |  |  |
|  |  |  |  |  |  | Combined Survivors | -0.47 | 0.08 | 2.17E-08 | 0.06 | 71.57 |
|  |  |  |  |  |  | Community Controls | -1.10 | 0.27 | 7.74E-05 |  |  |
|  | rs1963141 | 1 | 169672590 | G | A | SJLIFE1 Survivors | 0.42 | 0.10 | 9.39E-06 |  |  |
|  |  |  |  |  |  | SJLIFE2 Survivors | 0.76 | 0.19 | 7.87E-05 |  |  |
|  |  |  |  |  |  | Combined Survivors | 0.49 | 0.09 | 8.84E-09 | 0.11 | 60.22 |
|  |  |  |  |  |  | Community Controls | 1.08 | 0.27 | 9.69E-05 |  |  |
|  | rs7544874 | 1 | 169673308 | G | A | SJLIFE1 Survivors | 0.42 | 0.10 | 8.98E-06 |  |  |
|  |  |  |  |  |  | SJLIFE2 Survivors | 0.77 | 0.19 | 6.90E-05 |  |  |
|  |  |  |  |  |  | Combined Survivors | 0.49 | 0.09 | 7.71E-09 | 0.11 | 61.32 |
|  |  |  |  |  |  | Community Controls | 1.10 | 0.27 | 7.31E-05 |  |  |
|  | rs11407937 | 1 | 169673394 | insA | - | SJLIFE1 Survivors | 0.42 | 0.10 | 1.22E-05 |  |  |
|  |  |  |  |  |  | SJLIFE2 Survivors | 0.77 | 0.19 | 7.54E-05 |  |  |
|  |  |  |  |  |  | Combined Survivors | 0.49 | 0.09 | 1.18E-08 | 0.10 | 62.19 |
|  |  |  |  |  |  | Community Controls | 1.13 | 0.28 | 5.55E-05 |  |  |
|  | rs7537814 | 1 | 169674563 | A | T | SJLIFE1 Survivors | 0.45 | 0.09 | 1.59E-06 |  |  |
|  |  |  |  |  |  | SJLIFE2 Survivors | 0.77 | 0.19 | 7.29E-05 |  |  |
|  |  |  |  |  |  | Combined Survivors | 0.51 | 0.08 | 1.22E-09 | 0.13 | 55.42 |
|  |  |  |  |  |  | Community Controls | 1.09 | 0.28 | 1.01E-04 |  |  |
|  | rs7525857 | 1 | 169674567 | T | C | SJLIFE1 Survivors | 0.45 | 0.09 | 1.59E-06 |  |  |
|  |  |  |  |  |  | SJLIFE2 Survivors | 0.77 | 0.19 | 7.29E-05 |  |  |
|  |  |  |  |  |  | Combined Survivors | 0.51 | 0.08 | 1.22E-09 | 0.13 | 55.42 |
|  |  |  |  |  |  | Community Controls | 1.09 | 0.28 | 1.01E-04 |  |  |
|  | rs6690763 | 1 | 169676313 | A | T | SJLIFE1 Survivors | 0.43 | 0.10 | 7.87E-06 |  |  |
|  |  |  |  |  |  | SJLIFE2 Survivors | 0.77 | 0.19 | 6.90E-05 |  |  |
|  |  |  |  |  |  | Combined Survivors | 0.49 | 0.09 | 6.62E-09 | 0.11 | 60.76 |
|  |  |  |  |  |  | Community Controls | 1.09 | 0.27 | 9.29E-05 |  |  |
|  | rs6678930 | 1 | 169677023 | C | G | SJLIFE1 Survivors | 0.42 | 0.10 | 9.34E-06 |  |  |
|  |  |  |  |  |  | SJLIFE2 Survivors | 0.77 | 0.19 | 6.88E-05 |  |  |
|  |  |  |  |  |  | Combined Survivors | 0.49 | 0.09 | 8.08E-09 | 0.11 | 61.60 |
|  |  |  |  |  |  | Community Controls | 1.07 | 0.27 | 1.08E-04 |  |  |
|  | rs1011267 | 1 | 169677720 | G | A | SJLIFE1 Survivors | 0.43 | 0.09 | 5.02E-06 |  |  |
|  |  |  |  |  |  | SJLIFE2 Survivors | 0.76 | 0.19 | 8.07E-05 |  |  |
|  |  |  |  |  |  | Combined Survivors | 0.50 | 0.08 | 4.45E-09 | 0.13 | 57.30 |
|  |  |  |  |  |  | Community Controls | 1.09 | 0.27 | 8.94E-05 |  |  |
| EEAA-Hannum | rs2596483 | 6 | 31395903 | G | A | SJLIFE1 Survivors | -0.92 | 0.19 | 2.34E-06 |  |  |
|  |  |  |  |  |  | SJLIFE2 Survivors | -0.96 | 0.33 | 4.05E-03 |  |  |
|  |  |  |  |  |  | Combined Survivors | -0.93 | 0.17 | 2.94E-08 | 0.91 | 0 |
|  |  |  |  |  |  | Community Controls | -0.15 | 0.44 | 7.25E-01 |  |  |
|  | rs28366133 | 6 | 31396299 | T | C | SJLIFE1 Survivors | -0.93 | 0.18 | 3.74E-07 |  |  |
|  |  |  |  |  |  | SJLIFE2 Survivors | -1.17 | 0.31 | 2.17E-04 |  |  |
|  |  |  |  |  |  | Combined Survivors | -0.99 | 0.16 | 3.38E-10 | 0.51 | 0 |
|  |  |  |  |  |  | Community Controls | -0.34 | 0.39 | 3.89E-01 |  |  |

Abbreviations: GWAS, genome-wide association study; EAA, epigenetic age acceleration; Chr, chromosome; Pos_hg38, position in the homo sapiens (human) genome assembly GRCh38; SE, standard error; SJLIFE1 Survivors, the first discovery data set of 2138 survivors included in our previously published study (ref. 6); SJLIFE2 Survivors, the second discovery data set of 502 children and adolescent survivors; Combined Survivors, a combined set (meta-GWAS analysis) of the two discovery data sets (SJLIFE1 and SJLIFE2); Community Controls, a set of 282 community controls.

**Table S3. Differentially methylated regions (DMR) between the SJLIFE1 data set of survivors and controls overlapping with rs28366133 (+/- 500 kb) in *HLA* region.**

| **Chr** | **Start_hg19** | **End_hg19** | **Width** | **No.CpGs** | **Min_smoothed_FDR** | **Stouffer** | **HMFDR** | **Fisher** | **Maxdiff** | **Meandiff** | **Overlapping genes** |
| --- | --- | --- | --- | --- | --- | --- | --- | --- | --- | --- | --- |
| chr6 | 30874989 | 30886665 | 11677 | 90 | 9.98E-147 | 6.94E-155 | 5.27E-43 | 3.57E-287 | 5.65E-02 | 1.08E-03 | GTF2H4, VARS2 |
| chr6 | 30898937 | 30899405 | 469 | 3 | 2.16E-17 | 6.94E-12 | 1.60E-12 | 3.57E-14 | 1.37E-02 | 6.06E-03 | SFTA2 |
| chr6 | 30908302 | 30908739 | 438 | 4 | 2.81E-14 | 1.95E-10 | 9.76E-07 | 8.71E-12 | 1.62E-02 | 7.23E-03 | - |
| chr6 | 30909928 | 30910941 | 1014 | 7 | 7.73E-10 | 1.77E-05 | 9.62E-05 | 7.37E-07 | 8.27E-03 | 2.45E-03 | DPCR1 |
| chr6 | 30950282 | 30951917 | 1636 | 9 | 5.04E-15 | 1.00E-07 | 1.89E-07 | 1.90E-11 | 9.73E-03 | 4.32E-03 | MUC21 |
| chr6 | 30972851 | 30973562 | 712 | 4 | 3.49E-25 | 2.76E-11 | 7.91E-17 | 1.25E-19 | -1.26E-02 | -4.91E-03 | - |
| chr6 | 31021134 | 31022286 | 1153 | 9 | 2.21E-25 | 7.06E-14 | 8.04E-11 | 4.51E-19 | -9.22E-03 | -3.62E-03 | HCG22 |
| chr6 | 31025759 | 31027005 | 1247 | 5 | 8.51E-16 | 1.59E-10 | 2.55E-09 | 8.18E-14 | -2.07E-02 | -4.32E-03 | HCG22 |
| chr6 | 31038713 | 31039180 | 468 | 3 | 3.50E-08 | 2.17E-05 | 1.06E-04 | 1.62E-06 | 1.12E-02 | 7.49E-03 | - |
| chr6 | 31047558 | 31047822 | 265 | 7 | 8.88E-09 | 2.38E-05 | 1.13E-03 | 4.33E-07 | 3.90E-03 | 2.18E-03 | - |
| chr6 | 31096861 | 31097078 | 218 | 4 | 1.65E-16 | 4.17E-08 | 2.67E-11 | 6.94E-14 | 1.07E-02 | 5.70E-03 | PSORS1C1 |
| chr6 | 31110228 | 31111270 | 1043 | 5 | 6.55E-66 | 1.15E-25 | 7.11E-49 | 3.56E-51 | -2.18E-02 | -3.82E-03 | CCHCR1 |
| chr6 | 31112957 | 31113026 | 70 | 3 | 1.82E-17 | 1.36E-12 | 2.49E-13 | 1.31E-15 | -8.95E-03 | -5.78E-03 | CCHCR1 |
| chr6 | 31129244 | 31130516 | 1273 | 16 | 9.06E-29 | 5.93E-13 | 2.32E-09 | 1.88E-21 | 1.23E-02 | 1.90E-03 | TCF19 |
| chr6 | 31138420 | 31139796 | 1377 | 7 | 1.86E-14 | 3.36E-14 | 3.82E-07 | 1.03E-15 | 1.14E-02 | 4.90E-03 | POU5F1 |
| chr6 | 31148332 | 31148748 | 417 | 14 | 9.64E-43 | 4.70E-25 | 6.34E-14 | 5.94E-36 | 2.65E-02 | 9.16E-03 | POU5F1 |
| chr6 | 31168943 | 31169214 | 272 | 3 | 2.85E-09 | 1.28E-02 | 7.13E-08 | 2.63E-06 | 1.50E-02 | 4.91E-03 | HCG27, XXbac-BPG299F13.14 |
| chr6 | 31408964 | 31409757 | 794 | 9 | 1.78E-12 | 2.28E-10 | 1.21E-05 | 4.78E-10 | -1.09E-02 | 1.83E-03 | HCP5, LINC01149 |
| chr6 | 31473513 | 31478830 | 5318 | 17 | 3.09E-63 | 6.39E-42 | 5.52E-25 | 1.14E-71 | 3.38E-02 | 5.33E-03 | MICB |
| chr6 | 31495090 | 31497756 | 2667 | 12 | 3.70E-29 | 2.97E-16 | 1.90E-14 | 7.50E-27 | 1.77E-02 | 1.42E-03 | RPL15P4, MCCD1 |
| chr6 | 31502386 | 31503256 | 871 | 12 | 3.19E-50 | 1.20E-30 | 3.85E-16 | 2.25E-45 | -1.14E-02 | -2.20E-03 | DDX39B, ATP6V1G2-DDX39B |
| chr6 | 31507316 | 31511590 | 4275 | 42 | 1.07E-89 | 2.91E-60 | 2.42E-27 | 1.49E-116 | 2.82E-02 | 1.60E-03 | DDX39B-AS1, DDX39B, ATP6V1G2-DDX39B, SNORD84 |
| chr6 | 31512618 | 31516059 | 3442 | 30 | 4.01E-78 | 1.87E-50 | 1.46E-14 | 1.24E-75 | -2.39E-02 | -1.90E-03 | NFKBIL1, ATP6V1G2-DDX39B, ATP6V1G2 |
| chr6 | 31527889 | 31528239 | 351 | 4 | 6.63E-10 | 1.01E-04 | 5.13E-07 | 6.96E-07 | -1.72E-02 | -2.05E-03 | - |
| chr6 | 31542556 | 31543686 | 1131 | 16 | 6.22E-55 | 1.29E-35 | 5.94E-17 | 3.94E-45 | 1.87E-02 | -5.38E-03 | TNF |
| chr6 | 31545836 | 31551425 | 5590 | 39 | 1.36E-130 | 6.88E-103 | 4.44E-52 | 3.02E-190 | 2.20E-02 | 2.81E-03 | TNF, LTB |
| chr6 | 31554199 | 31555016 | 818 | 7 | 4.97E-11 | 6.79E-07 | 8.46E-06 | 4.22E-08 | -9.89E-03 | -4.43E-03 | LST1 |
| chr6 | 31608321 | 31609116 | 796 | 3 | 4.13E-12 | 2.62E-08 | 7.08E-07 | 2.12E-09 | -5.85E-03 | -4.42E-03 | BAG6 |
| chr6 | 31612351 | 31613356 | 1006 | 5 | 1.95E-15 | 6.38E-12 | 7.40E-09 | 1.72E-12 | -1.04E-02 | -5.54E-03 | BAG6 |
| chr6 | 31615385 | 31617606 | 2222 | 17 | 1.27E-13 | 6.67E-14 | 1.87E-06 | 6.44E-18 | 1.61E-02 | 3.45E-03 | BAG6 |
| chr6 | 31645405 | 31649728 | 4324 | 46 | 8.95E-126 | 1.43E-100 | 6.10E-22 | 1.55E-145 | 2.38E-02 | -2.16E-03 | LY6G5C |
| chr6 | 31650735 | 31651676 | 942 | 20 | 1.62E-29 | 3.29E-16 | 1.21E-09 | 1.68E-23 | -3.85E-02 | -8.76E-03 | LY6G5C |
| chr6 | 31652845 | 31652969 | 125 | 3 | 4.28E-14 | 1.53E-07 | 1.31E-11 | 1.37E-11 | 8.90E-03 | 2.28E-03 | - |
| chr6 | 31657467 | 31658176 | 710 | 4 | 9.11E-13 | 5.11E-09 | 7.15E-08 | 7.94E-11 | -8.18E-03 | -4.12E-03 | ABHD16A, XXbac-BPG32J3.20 |
| chr6 | 31680112 | 31680228 | 117 | 3 | 1.31E-18 | 7.82E-13 | 5.31E-14 | 1.03E-15 | -1.46E-02 | -9.65E-03 | LY6G6F, MEGT1, XXbac-BPG32J3.20, LY6G6E |
| chr6 | 31681562 | 31683075 | 1514 | 7 | 2.69E-24 | 2.08E-21 | 3.69E-09 | 3.89E-21 | 2.44E-02 | 5.78E-03 | LY6G6F, MEGT1, XXbac-BPG32J3.20, LY6G6E |
| chr6 | 31685430 | 31685575 | 146 | 6 | 3.68E-12 | 2.14E-06 | 1.49E-06 | 6.87E-10 | 1.21E-02 | 4.22E-03 | LY6G6F, MEGT1, LY6G6D |
| chr6 | 31695027 | 31699709 | 4683 | 62 | 2.41E-245 | 4.47E-180 | 1.43E-38 | 0.00E+00 | 4.50E-02 | 1.87E-03 | DDAH2, CLIC1 |
| chr6 | 31702774 | 31708299 | 5526 | 51 | 8.39E-143 | 5.26E-115 | 3.91E-52 | 8.50E-196 | -1.83E-02 | -1.85E-03 | MSH5, MSH5-SAPCD1, CLIC1 |
| chr6 | 31727279 | 31736112 | 8834 | 39 | 4.98E-58 | 3.01E-88 | 6.50E-16 | 2.36E-106 | 1.84E-02 | 1.16E-03 | MSH5, MSH5-SAPCD1, SAPCD1, SAPCD1-AS1, VWA7 |
| chr6 | 31737254 | 31737885 | 632 | 3 | 1.25E-23 | 4.94E-15 | 3.17E-16 | 9.48E-19 | -1.74E-02 | -9.63E-03 | VWA7 |
| chr6 | 31739837 | 31741184 | 1348 | 11 | 2.89E-14 | 2.43E-09 | 2.41E-07 | 3.40E-11 | 1.13E-02 | 1.93E-03 | VWA7 |
| chr6 | 31759412 | 31761055 | 1644 | 21 | 2.14E-51 | 1.90E-26 | 7.36E-12 | 2.66E-41 | 2.04E-02 | 4.43E-03 | VARS |
| chr6 | 31762409 | 31765634 | 3226 | 40 | 2.40E-192 | 3.94E-126 | 2.67E-24 | 4.32E-177 | -1.34E-02 | -2.43E-03 | VARS, LSM2 |
| chr6 | 31770844 | 31775893 | 5050 | 27 | 2.42E-153 | 4.19E-73 | 1.01E-74 | 2.80E-154 | 3.21E-02 | 4.91E-03 | LSM2 |
| chr6 | 31794278 | 31795678 | 1401 | 17 | 2.79E-37 | 1.26E-20 | 1.19E-18 | 4.74E-32 | -8.77E-03 | -2.39E-03 | HSPA1B |
| chr6 | 31797821 | 31799404 | 1584 | 7 | 1.04E-47 | 2.80E-33 | 8.77E-33 | 1.08E-58 | 4.26E-02 | 3.65E-03 | HSPA1B |
| chr6 | 31801042 | 31805329 | 4288 | 42 | 9.56E-118 | 8.19E-65 | 7.19E-31 | 7.34E-126 | 2.48E-02 | -1.51E-03 | C6orf48, SNORD48, SNORD52 |
| chr6 | 31842575 | 31843465 | 891 | 5 | 4.34E-12 | 2.14E-09 | 9.37E-07 | 4.94E-10 | 1.01E-02 | 2.53E-03 | SLC44A4 |
| chr6 | 31862350 | 31870990 | 8641 | 94 | 4.06E-236 | 2.20E-171 | 2.77E-29 | 1.46E-313 | 4.25E-02 | 1.10E-03 | C2, EHMT2, ZBTB12 |

**Abbreviations:** Chr, chromosome; No.CpGs, number of constituent CpG sites of DMR; Min_smoothed_FDR, Minimum FDR of the smoothed estimate; Stouffer, Stouffer summary transform of the individual CpG FDRs; HMFDR, Harmonic mean of the individual CpG FDRs; Fisher, Fisher combined probability transform of the individual CpG FDRs; Maxdiff, Maximum differential/coefficient within the DMR; Meandiff, Mean differential/coefficient across the DMR. The DMRs were filtered by No.CpGs >= 3 and |Meandiff| >= 0.001.

**Table S4. Multivariable linear regression models for the two top SNPs.**

| Parameters | EAA-Horvath ~ rs732314 | | EAA-Hannum ~ rs28366133 | |
| --- | --- | --- | --- | --- |
|  | Effect size | *P* | Effect size | *P* |
| Additive SNP effect | 0.50 | 1.55E-07 | 0.76 | 5.30E-08 |
| Sample age | 0.62 | <1.0E-08 | 0.72 | <1.0E-08 |
| Brain cranium-RT | 0.25 | 0.12 | -0.03 | 0.89 |
| Chest-RT | 0.50 | 0.03 | 1.76 | 1.04E-07 |
| Abdominal/pelvic-RT | 1.05 | 5.39E-05 | 0.53 | 0.14 |
| Alkylators | 0.52 | 2.07E-03 | 0.50 | 0.03 |
| Anthracycline | -0.06 | 0.72 | -0.26 | 0.28 |
| Vincristine | -0.22 | 0.25 | -0.30 | 0.26 |
| Glucocorticoids | -0.29 | 0.10 | -0.08 | 0.76 |
| Platinum | -0.25 | 0.28 | -0.63 | 0.05 |
| Epipodophyllotoxins | 0.21 | 0.23 | 0.29 | 0.23 |
| Sex | -0.33 | 0.02 | -0.91 | 2.55E-06 |

Abbreviations: RT, radiation therapy.

**Table S5. Estimated SNP heritability of EAA based on each of four clock methods using single-trait LD Score Regression of the GWAS of the SJLIFE1 data set.**

| **EAA** | **SNP heritability** | **SE** |
| --- | --- | --- |
| Horvath | 0.33 | 0.20 |
| Hannum | 0.17 | 0.23 |
| PhenoAge | -0.04 | 0.20 |
| GrimAge | -0.01 | 0.20 |

**Table S6. Estimated genetic correlation between EAA-Horvath and other traits using LD Score Regression of the GWAS of the SJLIFE1 data set (*P*<0.05).**

| **Trait correlated with EAA-Horvath** | **PMID** | **Category** | **Ethnicity** | **r_g_** | **SE** | **Z** | ***P*** | **h^2^_obs** | **h^2^_obs_SE** | **h^2^_int** | **h^2^_int_SE** | **gcov_int** | **gcov_int_SE** |
| --- | --- | --- | --- | --- | --- | --- | --- | --- | --- | --- | --- | --- | --- |
| Heart rate | 23583979 | hematological | Mixed | 0.40 | 0.18 | 2.20 | 0.03 | 0.08 | 0.01 | 1.01 | 0.01 | -0.01 | 4.40E-03 |
| Creatinine | 27005778 | metabolites | European | -0.72 | 0.29 | -2.49 | 0.01 | 0.12 | 0.03 | 1.01 | 0.01 | 0.01 | 4.60E-03 |
| Glycoprotein acetyls; mainly a1-acid glycoprotein | 27005778 | metabolites | European | 0.64 | 0.30 | 2.15 | 0.03 | 0.10 | 0.03 | 0.99 | 0.01 | -0.01 | 4.20E-03 |
| Falls in the last year | 0 | ukbb | European | 0.30 | 0.15 | 2.08 | 0.04 | 0.03 | 2.10E-03 | 1.00 | 0.01 | -0.01 | 4.70E-03 |
| Pulse rate | 0 | ukbb | European | 0.29 | 0.14 | 2.02 | 0.04 | 0.14 | 0.01 | 1.02 | 0.01 | -0.01 | 0.01 |
| Ever highly irritable/argumentative for 2 days | 0 | ukbb | European | 0.42 | 0.21 | 1.99 | 0.05 | 0.05 | 0.01 | 1.00 | 0.01 | -0.01 | 4.90E-03 |
| Illness_ injury_ bereavement_ stress in last 2 years: Financial difficulties | 0 | ukbb | European | 0.32 | 0.15 | 2.19 | 0.03 | 0.03 | 2.20E-03 | 1.03 | 0.01 | -0.01 | 4.80E-03 |
| Vitamin and mineral supplements: Vitamin A | 0 | ukbb | European | 0.87 | 0.41 | 2.11 | 0.03 | 0.00 | 1.50E-03 | 1.00 | 0.01 | -0.01 | 4.60E-03 |
| Types of physical activity in last 4 weeks: Light DIY (eg: pruning_ watering the lawn) | 0 | ukbb | European | -0.30 | 0.15 | -2.02 | 0.04 | 0.04 | 2.30E-03 | 1.01 | 0.01 | 0.01 | 4.90E-03 |

Abbreviations: PubMed ID, the PubMed identification number for the study from which the GWAS summary statistics for the trait were derived; SE, the standard error of the genetic correlation estimate; Z, the Z score; *P*, the association *P*-value for the genetic correlation estimate; h^2^_obs, the observed heritability of the named trait, as calculated by LDSR; h^2^_obs_se, the standard error of the estimated h^2^_obs; h^2^_int, the single-trait LDSR intercept for the health-related trait; h^2^_int_se, the standard error for the estimated h^2^_int; gcov_int, the cross-trait LDSR intercept; gcov_int, the standard error for the gcov_int.

**Table S7. Evaluation of the known loci in the current study.**

| **EAA** | **SNP** | **Chr** | **Pos_hg38** | **A1** | **A2** | **SJLIFE1 survivors** | | | **SJLIFE2 survivors** | | |  | **Meta-analysis** | | | | |
| --- | --- | --- | --- | --- | --- | --- | --- | --- | --- | --- | --- | --- | --- | --- | --- | --- | --- |
|  |  |  |  |  |  | **N** | **Effect size** | **P** | **N** | **Effect size** | **P** | **Effect size** | | **P** | **P_het_** | **I^2^** |  |
| IEAA-Horvath | rs2038903 | 1 | 8856458 | T | G | 2135 | -0.04 | 7.08E-01 | 502 | -0.39 | 3.80E-02 | -0.11 | | 1.99E-01 | 9.34E-02 | 64.47 |  |
|  | rs12043492 | 1 | 38991334 | T | C | 2131 | 0.26 | 7.87E-03 | 500 | 0.27 | 1.70E-01 | 0.26 | | 2.75E-03 | 9.68E-01 | 0.00 |  |
|  | rs2275558 | 1 | 164559883 | A | G | 2136 | -0.28 | 1.66E-02 | 502 | 0.17 | 4.58E-01 | -0.19 | | 6.87E-02 | 8.41E-02 | 66.50 |  |
|  | rs6687517 | 1 | 169652823 | C | T | 2136 | 0.44 | 4.43E-06 | 502 | 0.76 | 9.01E-05 | 0.50 | | 4.13E-09 | 1.37E-01 | 54.80 |  |
|  | rs1011267 | 1 | 169677720 | G | A | 2137 | 0.43 | 5.02E-06 | 501 | 0.76 | 8.07E-05 | 0.50 | | 4.45E-09 | 1.26E-01 | 57.30 |  |
|  | rs7550821 | 1 | 207856602 | T | C | 2138 | -0.04 | 6.84E-01 | 502 | -0.16 | 4.52E-01 | -0.07 | | 4.82E-01 | 6.26E-01 | 0.00 |  |
|  | rs1726672 | 1 | 236356202 | T | C | 2134 | -0.28 | 6.88E-03 | 502 | -0.27 | 1.94E-01 | -0.28 | | 2.69E-03 | 9.63E-01 | 0.00 |  |
|  | rs4832646 | 2 | 16521131 | C | G | 2137 | -0.14 | 2.21E-01 | 502 | -0.32 | 1.45E-01 | -0.17 | | 7.93E-02 | 4.58E-01 | 0.00 |  |
|  | rs79111787 | 3 | 47674055 | C | T | 2138 | 0.16 | 7.30E-01 | 189 | 0.30 | 8.21E-01 | 0.17 | | 6.89E-01 | 9.19E-01 | 0.00 |  |
|  | rs79070372 | 3 | 128510481 | A | G | 2136 | 0.28 | 6.55E-02 | 502 | 0.25 | 4.21E-01 | 0.28 | | 4.46E-02 | 9.14E-01 | 0.00 |  |
|  | rs2492286 | 3 | 128617455 | T | G | 2136 | 0.30 | 2.09E-02 | 501 | 0.56 | 3.41E-02 | 0.35 | | 2.62E-03 | 3.69E-01 | 0.00 |  |
|  | rs388649 | 3 | 138777967 | T | A | 2131 | 0.27 | 4.49E-03 | 501 | 0.24 | 2.06E-01 | 0.26 | | 1.86E-03 | 8.99E-01 | 0.00 |  |
|  | rs6440667 | 3 | 150287063 | C | G | 2137 | 0.36 | 4.84E-03 | 502 | 0.53 | 3.95E-02 | 0.40 | | 5.73E-04 | 5.52E-01 | 0.00 |  |
|  | rs1047210 | 3 | 160436470 | C | A | 2137 | -0.03 | 7.88E-01 | 502 | 0.39 | 4.43E-02 | 0.06 | | 5.16E-01 | 5.38E-02 | 73.11 |  |
|  | rs11706810 | 3 | 160442133 | C | T | 2137 | -0.04 | 6.75E-01 | 502 | 0.39 | 4.35E-02 | 0.04 | | 6.04E-01 | 4.53E-02 | 75.05 |  |
|  | rs144317085 | 4 | 104884951 | T | A | 2138 | -0.54 | 3.65E-02 | 190 | -0.76 | 3.39E-01 | -0.56 | | 2.22E-02 | 7.89E-01 | 0.00 |  |
|  | rs2736100 | 5 | 1286401 | A | C | 2134 | -0.04 | 7.05E-01 | 501 | -0.04 | 8.31E-01 | -0.04 | | 6.64E-01 | 9.91E-01 | 0.00 |  |
|  | rs2736099 | 5 | 1287225 | A | G | 2136 | 0.01 | 9.38E-01 | 502 | 0.16 | 4.32E-01 | 0.04 | | 5.45E-01 | 8.30E-01 | 0.00 |  |
|  | rs7744541 | 6 | 18104469 | A | T | 2134 | 0.30 | 2.01E-03 | 501 | 0.51 | 1.13E-02 | 0.34 | | 9.81E-05 | 3.54E-01 | 0.00 |  |
|  | rs6915893 | 6 | 18110325 | T | C | 2132 | 0.29 | 3.54E-03 | 502 | 0.52 | 8.39E-03 | 0.33 | | 1.50E-04 | 2.82E-01 | 13.48 |  |
|  | rs143093668 | 6 | 18114015 | T | C | 2137 | -0.86 | 1.89E-04 | 501 | -1.35 | 1.38E-03 | -0.98 | | 1.38E-06 | 3.12E-01 | 2.10 |  |
|  | rs10949481 | 6 | 18120798 | T | A | 2138 | -0.73 | 1.45E-03 | 502 | -1.29 | 1.93E-03 | -0.86 | | 1.73E-05 | 2.32E-01 | 29.96 |  |
|  | rs10949483 | 6 | 18122275 | A | G | 2131 | 0.28 | 3.72E-03 | 501 | 0.52 | 9.51E-03 | 0.33 | | 1.75E-04 | 2.89E-01 | 11.04 |  |
|  | rs76244256 | 6 | 18140332 | T | C | 2138 | -0.80 | 5.67E-04 | 502 | -1.35 | 1.36E-03 | -0.93 | | 4.64E-06 | 2.52E-01 | 23.73 |  |
|  | rs73397619 | 6 | 25624572 | C | T | 2138 | 0.20 | 6.23E-02 | 502 | 0.06 | 7.88E-01 | 0.17 | | 7.49E-02 | 5.39E-01 | 0.00 |  |
|  | rs10447389 | 6 | 25642349 | A | G | 2137 | 0.20 | 6.48E-02 | 502 | 0.02 | 9.27E-01 | 0.16 | | 9.29E-02 | 4.40E-01 | 0.00 |  |
|  | rs4712953 | 6 | 25671618 | T | A | 2138 | 0.20 | 6.38E-02 | 502 | -0.02 | 9.08E-01 | 0.15 | | 1.12E-01 | 3.34E-01 | 0.00 |  |
|  | rs28780071* | 6 | 29954890 | T | C | NA | NA | NA | NA | NA | NA | NA | | NA | NA | NA |  |
|  | rs12666349 | 7 | 31688566 | C | T | 2138 | -0.33 | 6.82E-03 | 502 | 0.39 | 1.09E-01 | -0.19 | | 8.92E-02 | 8.07E-03 | 85.75 |  |
|  | rs55637147 | 11 | 57343922 | C | T | 2135 | -0.24 | 1.31E-02 | 501 | -0.32 | 9.56E-02 | -0.26 | | 2.96E-03 | 7.25E-01 | 0.00 |  |
|  | rs10778517 | 12 | 106947886 | G | T | 2137 | -0.12 | 2.08E-01 | 502 | 0.20 | 3.01E-01 | -0.06 | | 5.10E-01 | 1.36E-01 | 55.05 |  |
|  | rs10735418 | 12 | 106949598 | C | T | 2137 | -0.08 | 4.33E-01 | 502 | 0.17 | 3.79E-01 | -0.03 | | 7.54E-01 | 2.55E-01 | 22.73 |  |
|  | rs12903325 | 15 | 50061080 | G | T | 2136 | 0.10 | 3.47E-01 | 502 | -0.18 | 3.98E-01 | 0.05 | | 6.46E-01 | 2.38E-01 | 28.05 |  |
|  | rs34003787 | 16 | 73037482 | T | C | 2138 | 0.40 | 1.84E-02 | 502 | -0.01 | 9.84E-01 | 0.32 | | 3.56E-02 | 2.84E-01 | 13.02 |  |
|  | rs78781855 | 17 | 55025006 | G | T | 2135 | -0.29 | 1.27E-02 | 502 | -0.18 | 4.26E-01 | -0.27 | | 9.66E-03 | 6.89E-01 | 0.00 |  |
|  | rs62078811 | 17 | 55031815 | A | G | 2135 | -0.30 | 9.38E-03 | 502 | -0.16 | 5.01E-01 | -0.27 | | 8.64E-03 | 5.73E-01 | 0.00 |  |
|  | rs954794 | 18 | 44501960 | A | G | 2136 | 0.41 | 2.31E-03 | 502 | -0.30 | 2.69E-01 | 0.27 | | 2.63E-02 | 1.80E-02 | 82.14 |  |
|  | rs57941717 | 21 | 37001879 | T | G | 2136 | 0.16 | 1.59E-01 | 502 | -0.20 | 3.63E-01 | 0.09 | | 3.96E-01 | 1.47E-01 | 52.39 |  |
|  | rs75243280 | 22 | 17120576 | C | T | 2119 | 0.01 | 9.28E-01 | 497 | -0.06 | 7.80E-01 | 0.00 | | 9.66E-01 | 7.71E-01 | 0.00 |  |
| EEAA-Hannum | rs10937913 | 4 | 2750698 | A | G | 2098 | 0.11 | 5.37E-01 | 497 | -0.28 | 3.67E-01 | 0.01 | | 9.37E-01 | 2.76E-01 | 15.86 |  |
|  | rs71007656 | 10 | 37681918 | CGGCTG | C | 2131 | -0.06 | 7.18E-01 | 502 | -0.67 | 3.92E-02 | -0.21 | | 1.86E-01 | 1.03E-01 | 62.43 |  |
|  | rs1005277 | 10 | 37929331 | A | C | 2137 | 0.03 | 8.78E-01 | 502 | 0.46 | 1.89E-01 | 0.13 | | 4.39E-01 | 2.84E-01 | 13.04 |  |
| EAA-Hannum | rs93059 | 4 | 102547361 | A | G | 2135 | 0.10 | 4.73E-01 | 501 | 0.35 | 1.24E-01 | 0.17 | | 1.57E-01 | 3.46E-01 | 0.00 |  |
|  | rs3093956 | 6 | 31459190 | C | T | 2138 | 0.72 | 1.20E-04 | 502 | 0.30 | 3.71E-01 | 0.63 | | 1.42E-04 | 2.77E-01 | 15.30 |  |
|  | rs111731678 | 7 | 130733917 | A | C,T | 2108 | -0.15 | 3.95E-01 | 497 | -0.50 | 8.02E-02 | -0.24 | | 1.03E-01 | 2.86E-01 | 12.20 |  |
|  | rs10508861 | 10 | 37775141 | A | G | 2138 | -0.04 | 8.10E-01 | 502 | 0.17 | 5.43E-01 | 0.01 | | 9.26E-01 | 5.17E-01 | 0.00 |  |
|  | rs117932856 | 10 | 37875836 | A | T | 2138 | 0.17 | 6.27E-01 | 190 | -0.34 | 7.46E-01 | 0.12 | | 7.19E-01 | 6.45E-01 | 0.00 |  |
|  | rs4838595 | 10 | 48467204 | T | C | 2137 | -0.16 | 4.49E-01 | 502 | 0.05 | 8.84E-01 | -0.11 | | 5.64E-01 | 6.08E-01 | 0.00 |  |
|  | rs941997 | 10 | 96287079 | T | A | 2137 | -0.12 | 4.55E-01 | 499 | 0.23 | 4.28E-01 | -0.04 | | 7.94E-01 | 2.90E-01 | 10.56 |  |
|  | rs12417758 | 11 | 66308889 | C | T | 2133 | 0.33 | 1.85E-02 | 502 | 0.53 | 2.41E-02 | 0.38 | | 1.47E-03 | 4.56E-01 | 0.00 |  |
|  | rs34970912 | 16 | 73034264 | G | C | 2138 | 0.53 | 1.71E-01 | 190 | -0.93 | 3.67E-01 | 0.35 | | 3.33E-01 | 1.85E-01 | 43.11 |  |
| EAA-GrimAge | rs887466 | 6 | 31175734 | A | G | 2044 | -0.02 | 8.10E-01 | 501 | 0.08 | 5.90E-01 | 0.01 | | 9.44E-01 | 5.57E-01 | 0.00 |  |
|  | rs9386796 | 6 | 109297501 | T | C | 2047 | 0.15 | 9.19E-02 | 501 | 0.09 | 5.21E-01 | 0.13 | | 7.61E-02 | 7.40E-01 | 0.00 |  |
|  | rs17094148 | 10 | 99520522 | G | A | 2044 | 0.19 | 5.09E-02 | 501 | 0.12 | 4.72E-01 | 0.17 | | 4.07E-02 | 6.99E-01 | 0.00 |  |
|  | rs1045929 | 17 | 40019173 | T | C | NA | NA | NA | NA | NA | NA | NA | | NA | NA | NA |  |
| EAA-PhenoAge | rs752223 | 1 | 59967404 | A | G | 2137 | -0.03 | 9.27E-01 | 503 | -0.92 | 1.17E-01 | -0.20 | | 4.34E-01 | 1.72E-01 | 46.47 |  |
|  | rs678553 | 1 | 236362147 | C | T | 2131 | -0.41 | 1.64E-02 | 502 | -0.91 | 1.27E-02 | -0.51 | | 1.19E-03 | 2.18E-01 | 33.97 |  |
|  | rs62114564 | 2 | 16524812 | T | G | 2132 | -0.31 | 9.55E-02 | 502 | -0.15 | 6.93E-01 | -0.28 | | 9.41E-02 | 7.13E-01 | 0.00 |  |
|  | rs4294009 | 6 | 18105845 | G | T | 2135 | 0.25 | 1.29E-01 | 502 | 0.54 | 1.23E-01 | 0.30 | | 4.29E-02 | 4.42E-01 | 0.00 |  |
|  | rs75407001 | 6 | 18120062 | T | C | NA | NA | NA | NA | NA | NA | NA | | NA | NA | NA |  |
|  | rs1990053 | 7 | 44886297 | A | G | 2138 | 0.00 | 9.98E-01 | 502 | 0.24 | 4.49E-01 | 0.05 | | 7.39E-01 | 4.96E-01 | 0.00 |  |
|  | rs11253338 | 10 | 713619 | T | C | 2136 | -0.20 | 3.28E-01 | 503 | -0.08 | 8.42E-01 | -0.18 | | 3.32E-01 | 8.11E-01 | 0.00 |  |
|  | rs11190127 | 10 | 99512225 | A | C | 2117 | 0.13 | 4.24E-01 | 500 | 0.28 | 4.12E-01 | 0.16 | | 2.83E-01 | 6.89E-01 | 0.00 |  |
|  | rs73028070 | 11 | 122811127 | A | G | 2137 | 0.11 | 7.02E-01 | 503 | -0.73 | 2.28E-01 | -0.04 | | 8.70E-01 | 2.10E-01 | 36.45 |  |
|  | rs3829957 | 17 | 3475582 | T | C | 2136 | -0.16 | 4.26E-01 | 502 | -0.58 | 1.59E-01 | -0.24 | | 1.83E-01 | 3.56E-01 | 0.00 |  |
|  | rs116853700 | 17 | 57388934 | A | G | 2116 | 0.32 | 4.34E-01 | 188 | 1.72 | 1.49E-01 | 0.47 | | 2.24E-01 | 2.66E-01 | 19.03 |  |
|  | rs7228835 | 18 | 44389106 | C | G | 2138 | -0.06 | 7.87E-01 | 502 | -1.36 | 1.20E-02 | -0.28 | | 2.06E-01 | 2.80E-02 | 79.30 |  |

**Abbreviations:** GWAS, genome-wide association study; EAA, epigenetic age acceleration; Chr, chromosome; Pos_hg38, position in hg38; A1, effect allele; A2: other allele; N: number of samples; SJLIFE1 Survivors, the first discovery data set of 2138 survivors included in our previously published study (ref. 6); SJLIFE2 Survivors, the second discovery data set of 502 children and adolescent survivors; Meta-analysis, meta-GWAS analysis of a combined set of the two discovery data sets (SJLIFE1 and SJLIFE2); IEAA, intrinsic epigenetic age acceleration; EEAA, extrinsic epigenetic age acceleration. * the SNP is not available.

**Table S8. DMR between the survivors (SJLIFE1 data set) and controls overlapping with *TERT* gene region.**

| **Chr** | **Start_hg19** | **End_hg19** | **Width** | **No.CpGs** | **Min_smoothed_FDR** | **Stouffer** | **HMFDR** | **Fisher** | **Maxdiff** | **Meandiff** | **Overlapping genes** |
| --- | --- | --- | --- | --- | --- | --- | --- | --- | --- | --- | --- |
| chr5 | 1243714 | 1244925 | 1212 | 9 | 2.22E-60 | 5.64E-43 | 1.34E-18 | 7.28E-52 | 3.08E-02 | 2.36E-03 | SLC6A18 |
| chr5 | 1246795 | 1247290 | 496 | 3 | 1.28E-08 | 1.90E-07 | 1.37E-04 | 4.35E-07 | 7.12E-03 | -2.08E-03 | NA |
| chr5 | 1266145 | 1266639 | 495 | 3 | 2.76E-13 | 8.78E-06 | 8.80E-12 | 7.82E-11 | 6.24E-03 | 3.13E-03 | TERT |
| chr5 | 1268644 | 1271848 | 3205 | 17 | 1.17E-56 | 2.75E-39 | 1.56E-28 | 2.13E-63 | 2.76E-02 | 6.74E-03 | TERT |
| chr5 | 1277054 | 1280305 | 3252 | 11 | 3.17E-33 | 1.29E-36 | 1.12E-18 | 1.21E-54 | 1.22E-02 | 1.85E-03 | TERT |
| chr5 | 1288569 | 1289283 | 715 | 4 | 2.75E-33 | 3.85E-25 | 1.55E-17 | 6.73E-29 | 1.80E-02 | 7.85E-03 | TERT |
| chr5 | 1291888 | 1292339 | 452 | 3 | 7.99E-09 | 4.18E-04 | 1.64E-07 | 6.34E-07 | 1.36E-02 | 2.92E-03 | TERT |
| chr5 | 1294198 | 1296007 | 1810 | 11 | 4.79E-52 | 3.57E-24 | 3.07E-16 | 5.04E-40 | 3.27E-02 | 1.96E-03 | TERT |
| chr5 | 1298511 | 1301123 | 2613 | 10 | 9.96E-36 | 2.92E-21 | 2.96E-27 | 1.64E-34 | -1.31E-02 | -3.82E-03 | NA |

**Abbreviations:** SJLIFE1 data set, the first discovery data set of 2138 survivors included in our previously published study (ref. 6); Chr, chromosome; No.CpGs, number of constituent CpG sites of DMR; Min_smoothed_FDR, Minimum FDR of the smoothed estimate; Stouffer, Stouffer summary transform of the individual CpG FDRs; HMFDR, Harmonic mean of the individual CpG FDRs; Fisher, Fisher combined probability transform of the individual CpG FDRs; Maxdiff, Maximum differential/coefficient within the DMR; Meandiff, Mean differential/coefficient across the DMR. The DMRs were filtered by No.CpGs >= 3 and |Meandiff| >= 0.001.
